# Characterization of the Fe(III)-Tiron System in Solution through an Integrated Approach Combining NMR Relaxometric, Thermodynamic, Kinetic, and Computational Data

**DOI:** 10.1021/acs.inorgchem.2c04393

**Published:** 2023-03-02

**Authors:** Alessandro Nucera, Fabio Carniato, Zsolt Baranyai, Carlos Platas-Iglesias, Mauro Botta

**Affiliations:** † Dipartimento di Scienze e Innovazione Tecnologica, Università del Piemonte Orientale, Viale Teresa Michel 11, 15121 Alessandria, Italy; ‡ Magnetic Resonance Platform (PRISMA-UPO), Università del Piemonte Orientale, Viale Teresa Michel 11, 15121 Alessandria, Italy; § Bracco Research Centre, Bracco Imaging S.p.A., Via Ribes 5, Colleretto Giacosa, 10010 Turin, Italy; ∥ Departamento de Química Fundamental, Facultade de Ciencias, Universidade da Coruña, Campus da Zapateira-Rúa da Fraga 10, 15008 A Coruña, Spain

## Abstract

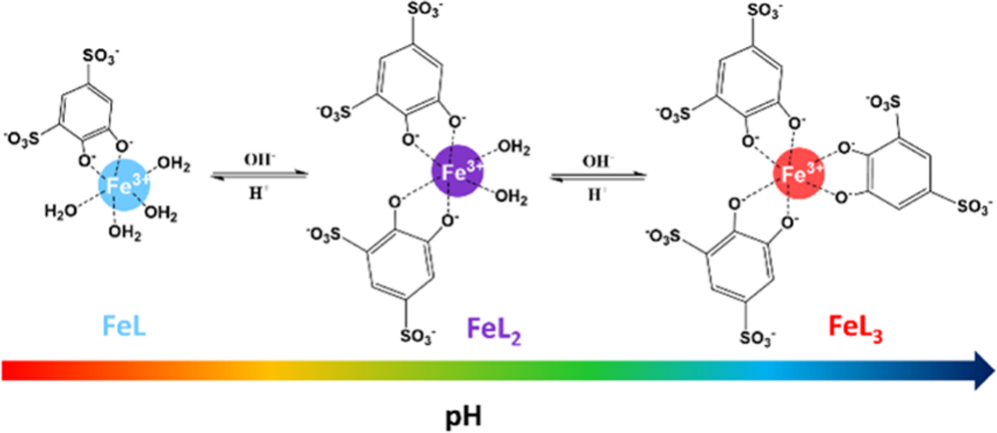

The Fe(III)-Tiron system (Tiron = 4,5-dihydroxy-1,3-benzenedisulfonate) was investigated using a combination of ^1^H and ^17^O NMR relaxometric studies at variable field and temperature and theoretical calculations at the DFT and NEVPT2 levels. These studies require a detailed knowledge of the speciation in aqueous solution at different pH values. This was achieved using potentiometric and spectrophotometric titrations, which afforded the thermodynamic equilibrium constants characterizing the Fe(III)-Tiron system. A careful control of the pH of the solution and the metal-to-ligand stoichiometric ratio allowed the relaxometric characterization of [Fe(Tiron)_3_]^9–^, [Fe(Tiron)_2_(H_2_O)_2_]^5–^, and [Fe(Tiron)(H_2_O)_4_]^−^ complexes. The ^1^H nuclear magnetic relaxation dispersion (NMRD) profiles of [Fe(Tiron)_3_]^9–^ and [Fe(Tiron)_2_(H_2_O)_2_]^5–^ complexes evidence a significant second-sphere contribution to relaxivity. A complementary ^17^O NMR study provided access to the exchange rates of the coordinated water molecules in [Fe(Tiron)_2_(H_2_O)_2_]^5–^ and [Fe(Tiron)(H_2_O)_4_]^−^ complexes. Analyses of the NMRD profiles and NEVPT2 calculations indicate that electronic relaxation is significantly affected by the geometry of the Fe^3+^ coordination environment. Dissociation kinetic studies indicated that the [Fe(Tiron)_3_]^9–^ complex is relatively inert due to the slow release of one of the Tiron ligands, while the [Fe(Tiron)_2_(H_2_O)_2_]^5–^ complex is considerably more labile.

## Abstract 2

The combined use of potentiometric data, ^1^H and ^17^O NMR relaxometric studies, and theoretical calculations enables a detailed characterization of pH-dependent Fe(III)-Tiron complexes, which differ in stoichiometry, hydration state, and overall electric charge.

## Introduction

It has long been known that chelators containing catechol functional groups play an important biological role. For example, the presence of this chemical moiety characterizes the siderophores, compounds involved in the bacterial Fe^3+^ sequestration.^1^ In fact, the ability of catechol ligands to coordinate stably various metal ions has been used in a number of therapies. In general, catechol chelators show a remarkable affinity toward metal ions in high oxidation states. In particular, disodium 4,5-dihydroxy-1,3-benzenedisulfonate (Tiron) is a water-soluble and nontoxic ligand capable of strongly coordinating different metal ions and therefore of potential interest for use in chelation therapy.^2^ In the chemical field, Tiron is known for analytical applications, primarily as a chelating agent used in the determination of trace metals. For example, Tiron is used as a colorimetric reagent of various metal ions, among which are Fe^3+^, Al^3+^, and Ti^4+^, for the sequestration of Pb^2+^, spectrofluorimetric determination of Cu^2+^, and spectrophotometric detection of Th^4+^ and Bi^3+^.^3−5^ More recently, it has been proposed for uses in electrochemistry concerning the preparation of redox flow batteries or modified glass electrodes.^6,7^

**Scheme 1:**
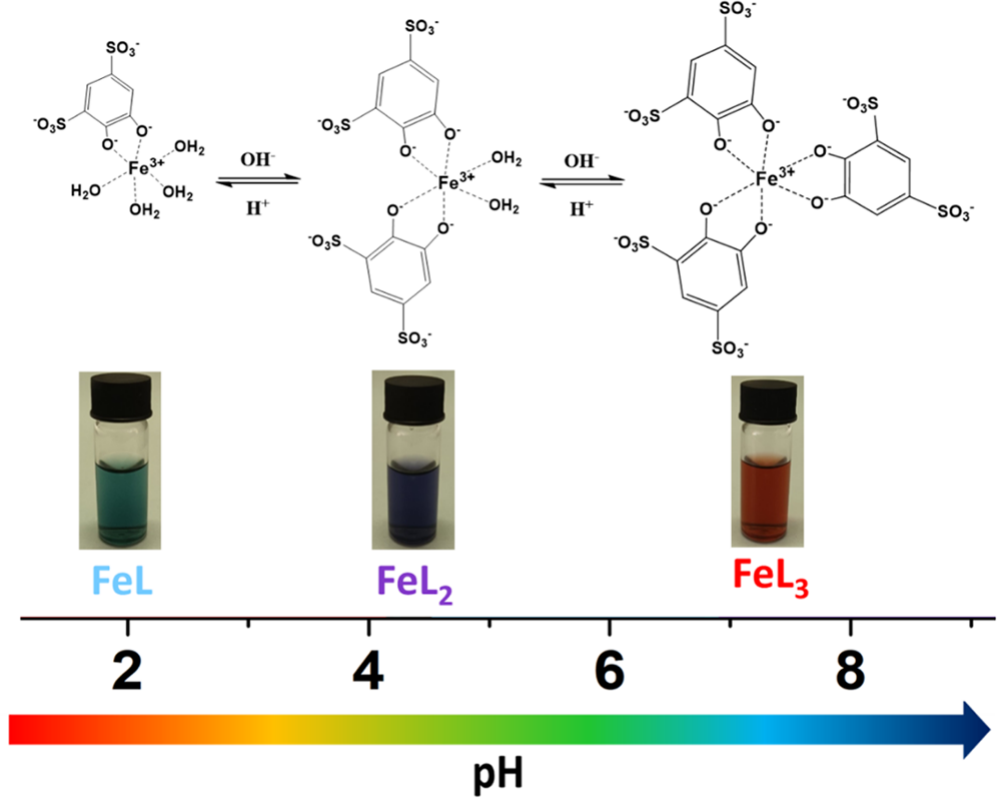
Different Complexes of the Fe(III)-Tiron System Present in Aqueous Solution as a Function of pH

However, Tiron is best known for its ability to form very stable Fe^3+^ complexes. This characteristic explains its widespread use as a complexometric indicator for the spectrophotometric detection of Fe^3+^ ions.^8−10^ The solution chemistry of the Fe(III)-Tiron system is quite complex and it is strongly affected by pH and ligand-to-metal molar ratios. As shown by UV–vis spectrophotometric data, three distinct coordination compounds can be identified in aqueous solution in different pH zones.^11,12^ Each species is characterized by a well-defined stoichiometry, which determines its state of hydration and therefore its reactivity, stability, and color. At pH values below 2, the turquoise-green solution is due to the presence of a complex in which only one unit of Tiron is coordinated to the metal center, which completes its coordination sphere with four water molecules (*q* = 4): [Fe(Tiron)(H_2_O)_4_]^−^. In the pH range of 4–5, the solution turns purple, indicating the coordination of a second Tiron with displacement of two Fe^3+^-bound water molecules and the formation of the bishydrated complex (*q* = 2): [Fe(Tiron)_2_(H_2_O)_2_]^5–^. Finally, above pH 7, the coordination sphere of the ferric ion is occupied by three bidentate Tiron ligands and therefore a *q* = 0 complex is present in the bright red solution: [Fe(Tiron)_3_]^9–^ (Scheme 1).

In the past, various studies have considered this system, and a number of data have been reported. However, detailed and complete characterization of each of the species present in solution is still missing. For instance, Ozutsumi et al. used EXAFS measurements to show that the three complexes share the same octahedral geometry characterized by Fe–O bond lengths of 200 pm.^13^ UV–vis spectrophotometry has been the main source of information on other physical–chemical properties of this system, such as thermodynamic stability, dissociation kinetics, and pH speciation.^11,12,14^ These early studies proved that all of the complexes of the Fe-Tiron system are thermodynamically extremely stable. The cumulative stability constants (log β values) of [Fe(Tiron)(H_2_O)_4_]^−^, [Fe(Tiron)_2_(H_2_O)_2_]^5–^, and [Fe(Tiron)_3_]^9–^ species are 18.7, 33.4, and 44.8, respectively. These three complexes are paramagnetic with high-spin iron and therefore their solutions are particularly suitable for NMR relaxometry studies. Fast-field-cycling relaxometry (FFC-NMR) consists in the investigation of the dependence of the longitudinal nuclear magnetic relaxation rate (1/*T*_1_ = *R*_1_) of the solvent protons on the applied magnetic field in a dilute solution of the solute. In the case of paramagnetic complexes, the analysis of these data, the so-called NMRD dispersion profiles, allows to accurately evaluate a series of important molecular parameters related to the structural and dynamic properties of the system. Among the most relevant, it is worth indicating the hydration number *q*, the distance *r*_MH_ between the metallic center and the protons of the coordinated water molecule, its average lifetime τ_M_ in the inner coordination sphere, the molecular reorientation rate of the complex 1/τ_R_, and the electronic relaxation times of the metal ion *T*_1,2e_.^15^ Fe^3+^, with five unpaired electrons in the 3d orbitals, a^6^S electronic ground state, and a high magnetic moment (μ_eff_ = 5.9 B.M.) is very well suited to be investigated through this technique.

In this work, we present a detailed ^1^H NMRD relaxation study of the Fe-Tiron system. Additional information on the exchange kinetics of the coordinated water molecule(s) is provided by the measurement and analysis of the ^17^O reduced transverse relaxation rates (*R*_2r_ = 1/*T*_2r_) and chemical shift (Δω_r_) of the bulk water as a function of temperature (280–350 K) at high field (11.74 T). To select the pH range in which the different complexes have a largely dominant population (>95%), an accurate species distribution diagram is required, which was obtained from potentiometric and spectrophotometric titration data. These data were accurately remeasured under conditions of ferric ions and Tiron concentrations suitable for the relaxometric study. Moreover, DFT computational calculations and spectrophotometric measurements were performed to obtain a complete and accurate description of the structural and dynamic characteristics of the species present in aqueous solutions of the Fe-Tiron system. Finally, to complete the study, we also evaluated their thermodynamic stability and kinetic inertness. We believe that the integrated use of these complementary techniques is able to provide a rather complete and accurate picture of the structure, dynamics, and properties of paramagnetic species in solution not easily accessible otherwise.

## Experimental Section

### Materials

For the relaxometric studies, a stock solution of Fe^3+^ was prepared from Fe(NO_3_)_3_·9H_2_O (reagent grade, Carlo Erba, Milano, Italy). The disodium 4,5-dihydroxy-1,3-benzenedisulfonate salt (Tiron) was reagent grade (Fluka AG, Buchs, Switzerland). For the thermodynamic and kinetic investigations, the chemicals used for the experiments were of the highest analytical grade. Fe(NO_3_)_3_ was prepared by dissolving Fe_2_O_3_ (99.9% Fluka) in 6 M HNO_3_ and evaporating of the excess acid. The solid Fe(NO_3_)_3_ was dissolved in a 0.1 M HNO_3_ solution. The concentration of the Fe(NO_3_)_3_ solution was determined by complexometry with the use of standardized Na_2_H_2_EDTA in excess. The excess of Na_2_H_2_EDTA was measured with a standardized ZnCl_2_ solution and xylenol orange as an indicator. The H^+^ concentration of the Fe(NO_3_)_3_ solution was determined by pH-potentiometric titration in the presence of Na_2_H_2_EDTA excess. The concentration of Tiron was determined by pH-potentiometric titration. All of the measurements were made at a constant ionic strength maintained by 0.15 M NaNO_3_ at 25 °C.

### Complexation of Iron(III)

For the synthesis of the Fe^3+^ chelates, 50 equiv of Tiron were dissolved in milliQ water, followed by the addition of 1 equiv of a Fe(NO_3_)_3_ stock solution (pH = 0.5, [Fe^3+^] = 14.45 mM, determined by the Evans’s method^16^). The pH was set to 2.51, 4.02, and 7.44 with NaOH 0.1 M to obtain [Fe(Tiron)(H_2_O)_4_]^−^, [Fe(Tiron)_2_(H_2_O)_2_]^5–^, and [Fe(Tiron)_3_]^9–^, respectively.

### Methods

#### Relaxometric Analysis

^1^H 1/*T*_1_ NMRD profiles were obtained with a fast-field-cycling Stelar SmartTracer relaxometer (Mede, Pavia, Italy) varying the magnetic-field strength from 0.00024 to 0.25 T (0.01–10 MHz range). The 1/*T*_1_ values are measured with an absolute uncertainty of ±1%. Temperature was controlled with a Stelar VTC-91 airflow heater equipped with a calibrated copper–constantan thermocouple (uncertainty of ±0.1 K). Data at high fields (0.5–3 T, corresponding to 20–120 MHz proton Larmor frequency) were collected with a High Field Relaxometer (Stelar) equipped with a HTS-110 3T Metrology Cryogen-free Superconducting Magnet. The measurements were performed with a standard inversion recovery sequence (20 experiments, two scans) with a typical 90° pulse width of 3.5 μs, and the reproducibility of the data was within ±0.5%. The Fe^3+^ concentration was estimated by ^1^H NMR (Bruker Advance III Spectrometer equipped with a wide bore 11.7 T magnet) measurements using Evans’s method.^16^

#### ^17^O NMR Measurements

The spectra were acquired on a Bruker Avance III spectrometer (11.7 T) using a 5 mm probe under temperature control. An aqueous solution of the complexes (≈8 mM for [Fe(Tiron)_2_(H_2_O)_2_]^5–^ and [Fe(Tiron)(H_2_O)_4_]^−^) was enriched to reach 2.0% of the ^17^O isotope (Cambridge Isotope). Transverse relaxation rates were measured from the signal width at half-height as a function of temperature, in the 278–350 K range for [Fe(Tiron)(H_2_O)_4_]^−^ and in the 278–310 K range for [Fe(Tiron)_2_(H_2_O)_2_]^5–^. The simultaneous fit of ^1^H NMRD profiles and ^17^O NMR data was performed with the Micromath Scientist computer program (version 2.0, Salt Lake City, Utah).

#### Equilibrium Measurements

The stability constant of [Fe(Tiron)*_x_*]^(4*x*−3)–^ species (*x* = 1–3) was determined by spectrophotometry, studying the Fe(III)-Tiron systems at the absorption band of the Fe^3+^ complex over the wavelength range of 400–800 nm in two sets of experiments. Individual samples were prepared in the first series in which the concentrations of Fe^3+^ and Tiron were constant at 0.19 and 9.1 mM, while that of H^+^ was varied between 0.01 and 1.0 mM (6 samples). The H^+^ concentration in the samples was adjusted by the addition of calculated amounts of 3.0 M HNO_3_. The ionic strength was constant in the samples with [H^+^] < 0.15 M ([H^+^] + [Na^+^] = 0.15 M). Samples were kept at 25 °C for a week. Absorbance values were determined at 11 wavelengths (425, 450, 475, 500, 550, 600, 625, 650, 700, 750, and 800 nm). In the second set, spectrophotometric titrations were done with samples containing the Tiron ligand in 9.1 mM concentration, whereas the concentration of Fe^3+^ was 0.19 mM. The pH of the samples was adjusted using concentrated NaOH and HNO_3_ solutions in the pH range of 2.0–10.0 (0.15 M NaNO_3_ and 25 °C). The stability and protonation constants of [Fe(Tiron)_3_]^9–^, [Fe(Tiron)_2_]^5–^, and [Fe(Tiron)_2_H_–1_]^6–^ complexes were determined by direct pH-potentiometric titration at 1:2 and 1:3 metal-to-ligand ratios (both concentrations were 0.002 M). For calculation of log *K*_FeL_3__, log *K*_FeL_2__, and log *K*_FeL_2_H_–1__ values, the mL base-pH data used were measured in the pH range of 1.7–12.0. For the calculation of the equilibrium constants, the best fit of the absorbance-pH and the mL base-pH data was obtained by assuming formation of [Fe(Tiron)]^−^, [Fe(Tiron)_2_]^5–^, [Fe(Tiron)_3_]^9–^, and [Fe(Tiron)_2_H_–1_]^6–^ species. The molar absorptivities of [Fe(Tiron)]^−^, [Fe(Tiron)_2_]^5–^, and [Fe(Tiron)_3_]^9–^ species were also determined at the same 11 wavelengths in these experiments. Spectrophotometric measurements were done using 1.0 cm cells with a PerkinElmer Lambda 365 UV–vis spectrophotometer at 25 °C.

#### pH Measurements and Titrations

A Metrohm 785 DMP Titrino titration workstation and a Metrohm-6.0233.100 combined electrode were used. Equilibrium measurements were carried out at a constant ionic strength (0.15 M NaNO_3_ or NaCl) in 6 mL samples at 25 °C. Solutions were stirred and continuously purged with N_2_. Titrations were performed in a pH range of 1.7–11.7. KH-phthalate (pH = 4.005) and borax (pH = 9.177) buffers were used to calibrate the pH meter. For calculation of [H^+^] from measured pH values, the method proposed by Irving et al. was used.^17^ A 0.01 M HNO_3_ solution was titrated with the standardized NaOH solution in the presence of 0.15 M NaNO_3_. Differences between the measured (pH_read_) and calculated pH (−log[H^+^]) values (pA) were used to obtain the equilibrium H^+^ concentration from the pH values, measured in the titration experiments (pA = 0.02). For equilibrium calculations, the stoichiometric water ionic product (p*K*_w_) is also needed to calculate [H^+^] values in basic conditions. The *V*_NaOH_–pH_read_ data pairs of HNO_3_–NaOH or HCl–NaOH titration obtained in the pH range of 10.5–12 have been used to calculate the p*K*_w_ value (p*K*_w_ = 13.77). For calculation of the equilibrium constants, the program PSEQUAD was used.^18^

#### Kinetic Studies

The kinetic inertness of the [Fe(Tiron)*_x_*]^(4*x*−3)–^ species (*x* = 2 and 3) was determined by the rates of CDTA (CDTA = *trans*-1,2-diaminocyclohexane-*N*,*N*,*N*′,*N*′-tetraacetic acid)-mediated ligand exchange reactions by spectrophotometry at 562 nm on the absorption band of [Fe(Tiron)*_x_*]^(4*x*−3)–^ in the pH range of 5.0–7.5. The total concentration of the [Fe(Tiron)*_x_*]^(4*x*−3)–^ complexes was 0.1 mM, while the concentration of CDTA was 20–80 times higher, to guarantee pseudo-first-order conditions. The temperature was maintained at 25 °C and the ionic strength of the solutions was kept constant using 0.15 M NaNO_3_. For keeping the pH values constant at pH = 5.0 and 5.5, 0.01 M piperazine buffers were used. At pH > 5.5, the buffer was not used since the CDTA excess was able to maintain a constant pH value due to the protonation equilibria between HCDTA and H_2_CDTA species (log *K*_2_^H^ = 6.08, 0.15 M NaNO_3_, 25 °C^19^). The pseudo-first-order rate constants (*k*_d_) were calculated by fitting the absorbance data to eq 1
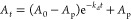
1where *A_t_*, *A*_0_, and *A*_p_ are the absorbance values at time *t*, the start of the reaction, and at equilibrium, respectively. The calculation of the kinetic parameters was performed by the fitting of the absorbance–time pairs with the Micromath Scientist computer program (version 2.0, Salt Lake City, Utah).

#### Computational Studies

The geometries of [Fe(Tiron)_3_]^9–^·5H_2_O, [Fe(Tiron)_2_(H_2_O)_2_]^5–^·9H_2_O, and [Fe(Tiron)(H_2_O)_4_]^5–^·13H_2_O systems were optimized at the uTPSSh/Def2-TZVPP^20,21^ level with the Gaussian16^22^ program package. All systems were modeled in their high-spin (sextet) configurations. The input geometries were generated from that reported previously^19^ for the [Fe(H_2_O)_6_]^3+^·12H_2_O cluster. The inclusion of explicit second-sphere water molecules is necessary for an accurate calculation of ^17^O hyperfine coupling constants.^23^ Bulk solvent effects were incorporated using a polarized continuum model [scrf = (pcm, solvent = water)].^24^ The integration grid was set with the integral = superfinegrid option. Frequency calculations were subsequently used to corroborate that the optimized geometries corresponded to stationary points on the potential energy surface.

Hyperfine coupling constants (*A*/*ℏ* = 2π × *a*^iso^) were calculated using the ORCA software package (5.0.3)^25,26^ at the uTPSSh/Def2-TZVPP level.^20,21^ These calculations incorporated the resolution of identity and chain of spheres approximation (RIJCOSX)^27,28^ with the aid of the Def2/J^29^ auxiliary basis set. Spin–orbit coupling effects were considered with the spin–orbit mean-field method [SOMF(1X)].^30^ The integration grids were increased from the defaults using the IntAcc 5.0 and AngularGrid 7 keywords. Zero-field splitting (ZFS) parameters were computed with *N*-electron valence perturbation theory to second order (SC-NEVPT2)^31,32^ on the top of complete active space self-consisted field (CASSCF)^33−35^ calculations with the Def2-TZVPP basis set. The active space consisted of five electrons distributed in the five metal-based 3dorbitals [CAS(5,5)], with the state-average CASSCF calculation incorporating one sextet, 24 quartet, and 75 doublet roots. All calculations were performed with the aid of the resolution of identity (RI) approximation for both Coulomb and exchange (RI-JK) using the Def2/JK auxiliary basis set.^29,36^ Bulk water solvent effects in all ORCA calculations were introduced with the SMD model developed by Truhlar,^37^ which is based on the electron density of the solute and a polarized continuum.

## Results and Discussion

### Solution Thermodynamics

The acid–base properties of the Tiron ligand were studied by pH potentiometry. The protonation constants (log *K*_i_^H^) of the ligand, defined by eq 2, are listed in Table 1 (standard deviations are shown in parentheses)
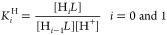
2The protonation scheme of Tiron was well characterized by both spectroscopic and potentiometric methods.^11,12^ These studies reveal that the first and second protonations of Tiron occur at two phenolate O-donor atoms.

**Table 1:** Protonation Constants of Tiron, Stability (log *K*_FeL*_x_*_), and Protonation Constants of the Corresponding Fe(III) Complexes (25 °C)

*I*	0.15 M NaNO_3_	0.2 M KCl^12^	1.0 M KNO_3_^11^
log *K*_1_^H^	12.40 (1)	11.96	11.78
log *K*_2_^H^	7.46 (3)	7.46	7.19
log *K*_FeL_	20.32 (1)	18.61	18.8
log *K*_FeL_2__	14.49 (2)	14.77	14.7
log *K*_FeL_3__	9.83 (2)	11.06	11.60
log *K*_FeL_2_H_–1__	7.86 (6)	5.98	

**Figure 1:**
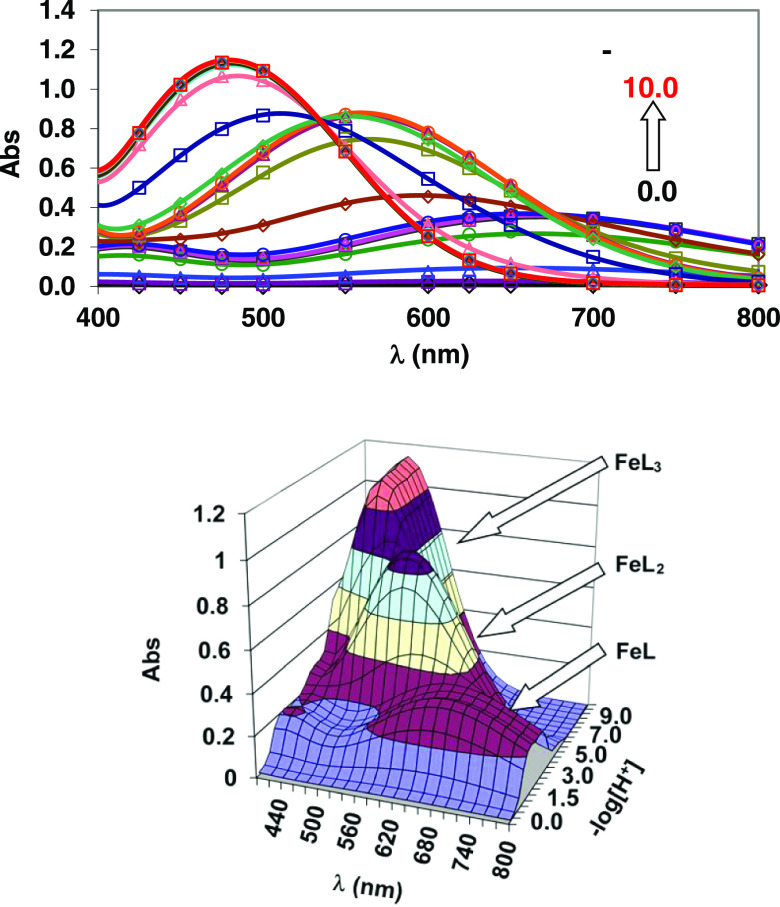
Absorption spectra of the Fe(III)-Tiron system. The solid lines and open symbols represent the experimental and calculated absorbance values, respectively ([Fe^3+^] = 0.19 mM, [Tiron] = 9.1 mM, −log[H^+^] = 0.0–10.0, 0.15 M NaNO_3_, 25°C).

**Figure 2:**
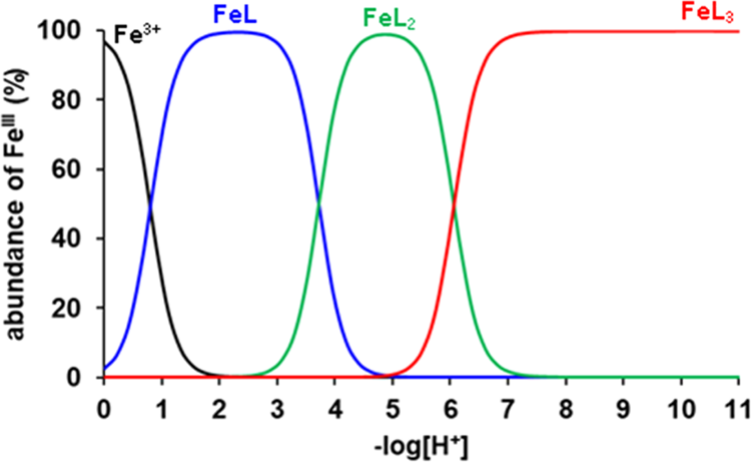
Species distribution of the Fe(III)-Tiron system ([Fe^3+^] = 0.2 mM, [L = Tiron] = 9.0 mM, 0.15 M NaNO_3_, 25 °C).

**Figure 3:**
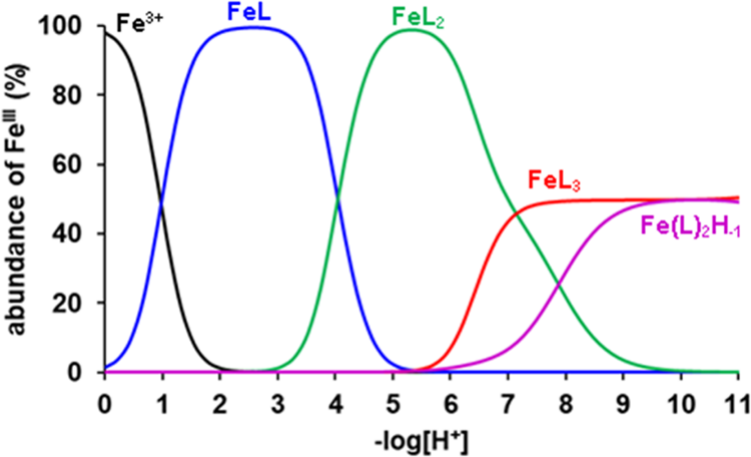
Species distribution of the Fe(III)-Tiron system ([Fe^3+^] = 0.2 mM, [L = Tiron] = 5.0 mM, 0.15 M NaNO_3_, 25 °C).

Comparison of the protonation constants obtained in 0.15 M NaNO_3_, 0.2 M KCl, or 1.0 M KNO_3_ indicates that log *K*_i_^H^ values of Tiron are independent of the ionic strength (Table 1). Tiron forms very stable complexes with the Fe^3+^ ion.^12^ Consequently, the determination of the equilibrium constants characterizing the species formed in the Fe(III)-Tiron system based only on pH-potentiometric studies is impossible. However, the interaction between the Fe^3+^ ion and Tiron can be studied by monitoring the charge transfer absorption band in the wavelength range of 400–800 nm. The absorption spectra of the Fe(III)-Tiron system recorded in the −log[H^+^] range 0.0–10.0 are shown in Figure 1. The spectral changes can be interpreted by the formation of [Fe(Tiron)]^−^ (Fe**L**_**1**,_ λ_max_ = 680 nm; eq 3), [Fe(Tiron)_2_]^5–^ (Fe**L**_**2**_, λ_max_ = 562 nm; eq 4), and [Fe(Tiron)_3_]^9–^ (Fe**L**_**3**_, λ_max_ = 480 nm; eq 5) species in the −log[H^+^] ranges of 0.0–3.0, 3.0–6.0, and 6.0–8.0, respectively
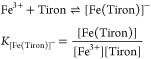
3
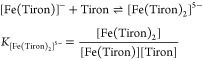
4
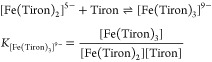
5The pH-potentiometric data of the Fe(III)-Tiron system at 1:2 metal-to-ligand concentration ratio indicate base consuming processes in the pH range of 6.0–9.0. These processes can be accounted for by the formation of [Fe(Tiron)_2_H_–1_]^6–^ species via the substitution of a H_2_O molecule with an OH^–^ anion (eq 6)
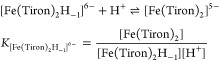
6The stability and protonation constants of [Fe(Tiron)]^−^, [Fe(Tiron)_2_]^5–^, [Fe(Tiron)_3_]^9–^, and [Fe(Tiron)_2_H_–1_]^6–^ species are shown in Table 1, while species distribution diagrams of the Fe(III)-Tiron system are presented in Figures 2, 3, and S1.

### Relaxometric Characterization

**Figure 4:**
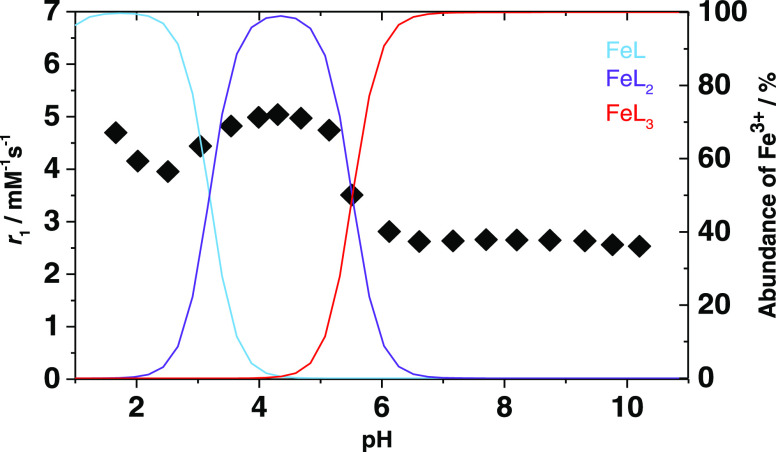
pH dependency of *r*_1_ for the Fe(III)-Tiron system (32 MHz and 298 K, 1:50 ratio). The solid lines correspond to the percentage abundance of each of the three species (L = Tiron).

1/*T*_1_ NMR relaxation measurements were performed as a function of pH, at 298 K and 32 MHz, to evaluate relaxivity and identify the pH range in which each species prevails (Figure 4). The ability to increase the relaxation rate is called relaxivity (*r*_1_), and it measures the relaxation rate enhancement of water proton nuclei normalized to a 1 mM concentration of the paramagnetic ion. The sample was prepared following a well-established procedure reported in the literature, using a Fe^3+^/Tiron molar ratio of 1:50.^38^ In the *r*_1_ vs pH profile of Figure 4, three different regions corresponding to the prevalence of different [Fe(Tiron)*_x_*]^(4*x*−3)–^ complexes are clearly distinguishable. The corresponding species distribution diagram is also shown in the same graph. The tetraaquo complex prevails in the acidic region (pH ≤ 2.2) and possesses an *r*_1_ value of 3.5 mM^–1^ s^–1^ at pH = 2.0. The [Fe(Tiron)_2_(H_2_O)_2_]^5–^ complex is largely prevalent (>90%) in the range of ca. 3.7 < pH < 4.9 and has an *r*_1_ value of 5.0 mM^–1^ s^–1^ at pH = 4.0. Finally, at pH values higher than 6.5, the aqueous solution contains only the outer-sphere (*q* = 0) complex [Fe(Tiron)_3_]^9–^ and *r*_1_ shows a constant value of ca. 2.9 mM^–1^ s^–1^. From a preliminary qualitative evaluation, it is quite evident that the relaxivity does not correlate directly with the hydration state of the complexes.

It is well-established that the relaxivity can be considered as the sum of three contributions describing the different ways in which the modulation of the dipolar coupling between the electron magnetic moment of the metal ion and the nuclear magnetic moment of the water protons can occur. One is associated with the water molecule(s) directly bound to the metal ion (inner sphere; IS), the other arises from water molecules interacting with polar groups of the ligand through long-lived hydrogen bonds (second sphere; SS), and the third due to bulk water molecules diffusing in the proximity of the paramagnetic complex (outer sphere, OS)
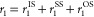
7The most important contribution is that associated with *r*_1_^IS^, which is directly proportional to the number of coordinated water molecules *q*. In fact, the inner-sphere relaxivity is given by the following expression^39^
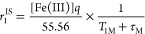
8In eq 8, *T*_1M_ is the relaxation rate of inner-sphere protons and τ_M_ (τ_M_ = 1/*k*_ex_) the mean residence time of a water molecule in the inner coordination sphere of the metal ion. The relaxation rate of inner-sphere protons in Fe^3+^ complexes is generally dominated by the dipole–dipole (DD) mechanism
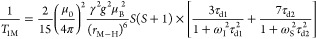
9In eq 9, *g* is the electron *g*-factor, *r*_M–H_ the distance between the electron and nuclear spins, *μ*_B_ the Bohr magneton, γ the proton gyromagnetic ratio, *S* the total spin (5/2 for a high-spin Fe^3+^ complex), ω_I_ the proton resonance frequency, and ω_S_ the Larmor frequency of the Fe^3+^ electron spin. The correlation time τ_d*i*_ is given by eq 10, where τ_R_ is the rotational correlation time and *T*_*i*e_ are the longitudinal (*i* = 1) and transverse (*i* = 2) relaxation times of the electron spin
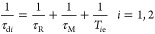
10*r*_1_^IS^ scales with *q* and therefore making the reasonable assumption of a comparable contribution of *r*_1_^OS^ in the three complexes, we should expect that *r*_1_ ([Fe(Tiron)(H_2_O)_4_]^−^) > *r*_1_ ([Fe(Tiron)_2_(H_2_O)_2_]^5–^) > *r*_1_ ([Fe(Tiron)_3_]^9–^). It follows that the lack of a clear relationship between *q* and *r*_1_ is connected to the occurrence of a significant contribution of *r*_1_^SS^. In particular, this mechanism appears to be dominant in the case of [Fe(Tiron)_3_]^9–^ and could be attributed to H-bonding interaction between water molecules and the six sulfonic groups on the three Tiron ligands.

#### [Fe(Tiron)_3_]^9–^

**Figure 5:**
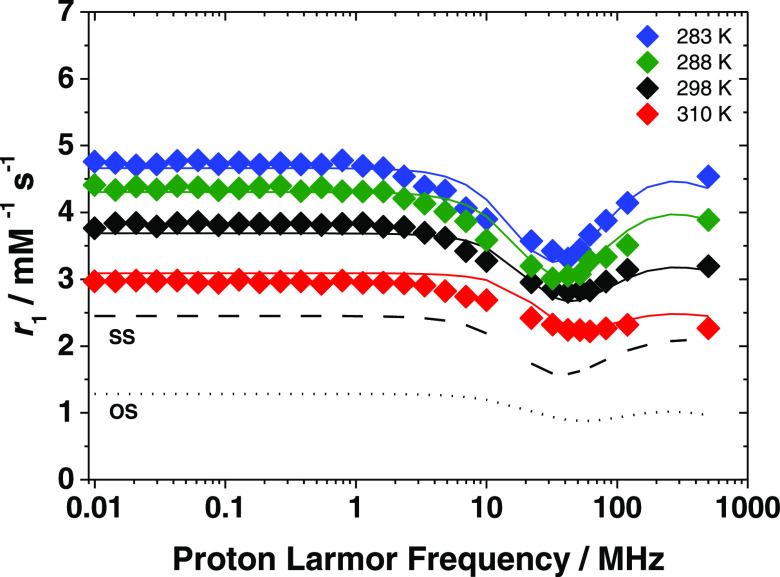
^1^H NMRD profiles of [Fe(Tiron)_3_]^9–^ at 283 (violet diamond solid), 288 (green diamond solid), 298 (black diamond solid), and 310 (red diamond solid) K and pH = 7.4 ([Fe^3+^] = 1.88 mM, [Tiron] = 94.0 mM). The solid lines correspond to the fit of the data. Dashed and dotted curves show the calculated second- and outer-sphere contributions to relaxivity, respectively (298 K).

This additional contribution can be easily appreciated by comparing the relaxation values for this complex and [Fe(DTPA)]^2–^, both *q* = 0 complexes. At 1.5 and 3.0 T (298 K), the *r*_1_ values are 2.8 and 3.1 mM^–1^ s^–1^ for [Fe(Tiron)_3_]^9–^ and 0.71 and 0.72 mM^–1^ s^–1^ for [Fe(DTPA)]^2–^. For [Fe(Tiron)_3_]^9–^, these *r*_1_ values are about 300% greater than the corresponding values for [Fe(DTPA)]^2–^. These values are also significantly greater than those reported for several other *q* = 0 complexes, such as [Fe(EHPG)]^−^, [Fe(EHBG)]^−^, [Fe(NOTA)]^−^, and their derivatives^19,40−46^ and comparable to those characterized by a SS contribution.^47,48^ The SS contribution to the relaxivity of [Fe(Tiron)_3_]^9–^ can be assessed by measuring the 1/*T*_1_^1^H NMRD profiles over a wide range of frequency values and analyzing the data using the Freed’s equation for the OS mechanism and eqs 8–10, suitable also for the SS mechanism by making some reasonable assumptions.^49,50^ The experimental profiles, shown in Figure 5, were measured over the proton Larmor frequency range of 0.01–500 MHz at four different temperatures (283, 288, 298, and 310 K) and at pH = 7.4. Typical values of the Fe^3+^ complexes were assigned to some parameters that describe the OS contribution: the diffusion coefficient (*D*), its activation energy (*E*_D_), and the distance of the closest approach (*a*) of the proton nuclei of outer-sphere water molecules to the paramagnetic ion (Table 2).^19^ For the fit of the data, also some of the parameters of the SS contribution were fixed to reasonable values. The number of SS water molecules was set to five (*q*^SS^ = 5), while their distance from the metal ion, *r*^SS^, was set to 3.5 Å. This assumption was based on DFT calculations performed on the [Fe(Tiron)_3_]^9–^·5H_2_O system (Figure S2), which contains three second-sphere water molecules hydrogen-bonded to the three negatively charged sulfonate groups that lie close to the *C*_3_ symmetry axis of the complex. Two additional second-sphere water molecules are involved in hydrogen bonds with the oxygen atoms of the phenolate groups on the opposite side. The remaining three sulfonate groups are rather far away from the metal center, and thus second-sphere water molecules bonded to these groups are not expected to provide a significant contribution to relaxivity, which depends on 1/*r*^6^. The second-sphere water molecules display *r*^SS^ distances in the range of 3.2–5.3 Å, with an average 1/*r*^6^ value that corresponds to *r* = 3.5 Å. The average life of the water molecules of the second-sphere τ_M_^SS^ is rather short and therefore such as not to influence relaxivity at any frequency value. Typically, τ_M_^SS^ is fixed at a value of 1 ns. The structure obtained with DFT is very similar to that determined by X-ray diffraction, which shows two sets of Fe–O bond distances of 1.950 and 2.109 Å with an average value of 2.03 Å.^51^ In the calculated structure, the Fe–O bond lengths vary in the range of 2.01–2.09 Å, with an averaged value of 2.05 Å. The latter value is in excellent agreement with that determined in solution with EXAFS measurements (2.04 Å).^13^

**Table 2:** Data Obtained from the Simultaneous Fit of ^1^H NMRD Profiles of ^17^O NMR Data

parameters	[Fe(Tiron)_3_]^9–^ (MW = 860 g mol^–1^)	[Fe(Tiron)_2_(H_2_O)_2_]^5–^ (MW = 628 g mol^–1^)	[Fe(Tiron)(H_2_O)_4_]^−^ (MW = 395 g mol^–1^)	[Fe(H_2_O)_6_]^3+^ (MW = 164 g mol^–1^)^b^
^298^*r*_1_ 60/120 MHz (mM^–1^ s^–1^)	2.8/3.2	5.4/6.5	3.4/3.3	12.9/14.1
^298^Δ^2^ (10^20^ s^–2^)	9.7 ± 0.1	12.2 ± 0.1	5.5 ± 0.1	4.2
*E*_Δ_ (kJ mol^–1^)	4.6 ± 0.9	2.7 ± 1.2	3.3 ± 1.7	
^298^τ_V_ (ps)	6.9 ± 0.1	5.6 ± 0.6	9.2 ± 0.5	5.3
*A*_O_/*ℏ* (10^6^ rad s^–1^)		–50.1 ± 4.8	–71.6 ± 5.7	–99.3^a^
^298^τ_M_^O^ (ns)		272 ± 57	18 000 ± 720	25 000
Δ*H*_M_ (kJ mol^–1^)		56.2 ± 10.7	57.5 ± 2.5	31.4
*C*_os_		0.04 ± 0.02	0.05 ± 0.01	0.038
^298^τ_R_ (ps)		70.0 ± 5.5	34.7 ± 2.5	60.7
*E*_R_ (kJ mol^–1^)		16.0 ± 4.9	15.0 ± 5.0	17.9
^298^τ_R_^SS^ (ps)	52.7 ± 0.2	31.2 ± 8.2		
*E*_R_^SS^ (kJ mol^–1^)	15.3 ± 0.6	11.0 ± 1.4		
*q*	0^a^	2^a^	4^a^	6^a^
*q*^SS^	5^a^	2^a^		
*r* (Å)		2.70^a^	2.70^a^	2.69^a^
*r*^SS^ (Å)	3.50^a^	3.25^a^		

a Fixed parameters. Additional parameters fixed during the best-fit procedure: *E*_V_ = 1.0 kJ mol^–1^, ^298^*D* = 2.24 × 10^5^ cm^2^ s^–1^, *E*_D_ = 20.0 *k*J mol^–1^, and *a* = 3.5 Å.

b From ref (19).

The best-fit parameters are reported in Table 2. We obtained an excellent fit of the *r*_1_ profiles of [Fe(Tiron)_3_]^9–^ on the basis of a τ_R_^SS^ of 52.7 ps and an associated activation energy, *E*_R_^SS^, of 15.3 kJ mol^–1^. The parameters characterizing the relaxation of the electron spin, the mean square transient ZFS energy (Δ^2^), and its correlation time (τ_v_) assume values in the normal range reported for Fe^3+^ chelates and fully comparable to those calculated for [Fe(CDTA)(H_2_O)]^−^.^19^ As previously observed, the variation of *r*_1_ with temperature is well reproduced if the zero-field splitting energy Δ is allowed to vary with temperature, according to an Arrhenius behavior with activation energy *E*_Δ_ (Table 2).^19^ From the calculated SS and OS contributions to the NMRD profile at 298 K (Figure 5), we can conclude that the *r*_1_^SS^ component represents a contribution of about 65–70% to total relaxivity.

#### [Fe(Tiron)_2_(H_2_O)_2_]^5–^

**Figure 6:**
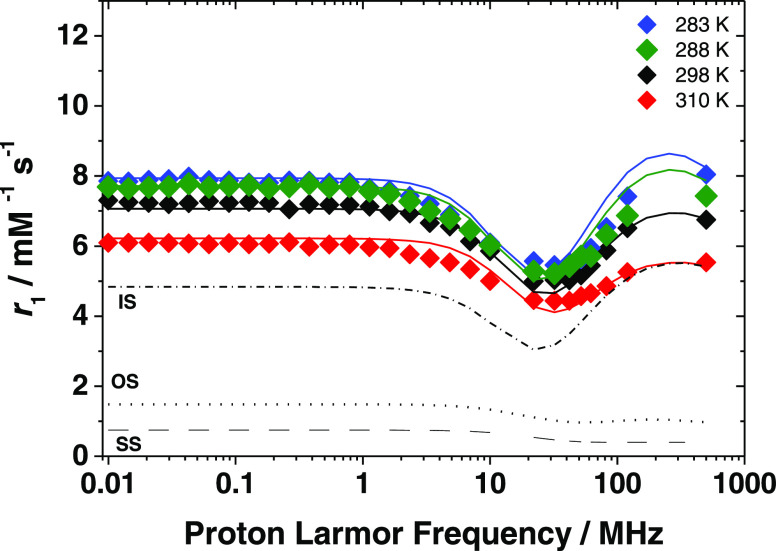
^1^H NMRD profiles (pH = 4.0) at different temperatures (283 (violet diamond solid), 288 (green diamond solid), 298 (black diamond solid), and 310 K (red diamond solid)) of [Fe(Tiron)_2_(H_2_O)_2_]^5–^ ([Fe^3+^] = 1.88 mM, [Tiron] = 94.0 mM). The solid lines correspond to the fit of the data. Dot–dashed, dashed, and dotted curves show the calculated inner-, second-, and outer-sphere contributions to relaxivity at 298 K.

As previously mentioned, the value of *r*_1_ of [Fe(Tiron)_2_(H_2_O)_2_]^5–^, at 32 MHz and 298 K, is 5.0 mM^–1^ s^–1^, which is markedly higher than that of the *q* = 1 complex [Fe(CDTA)(H_2_O)]^−^ (*r*_1_ = 2.2 mM^–1^ s^–1^) under similar experimental conditions.^19^ This simple consideration highlights the lack of a clear relationship between the relaxivity and hydration state, suggesting also in this case the presence of a marked contribution of the SS mechanism. The NMRD profiles are reported in Figure 6 and need to be analyzed taking into account all three contributions to *r*_1_. DFT calculations suggest the presence of two second-sphere water molecules at a close distance with respect to the paramagnetic center, which led us to assume the presence of two water molecules (*q*^SS^ = 2) and a *r*^SS^ value of 3.25 Å (Figure S3). Furthermore, with regard to the analysis of SS and OS contributions, we used the same procedure described for [Fe(Tiron)_3_]^9–^, setting different parameters at reasonable values.

**Figure 7:**
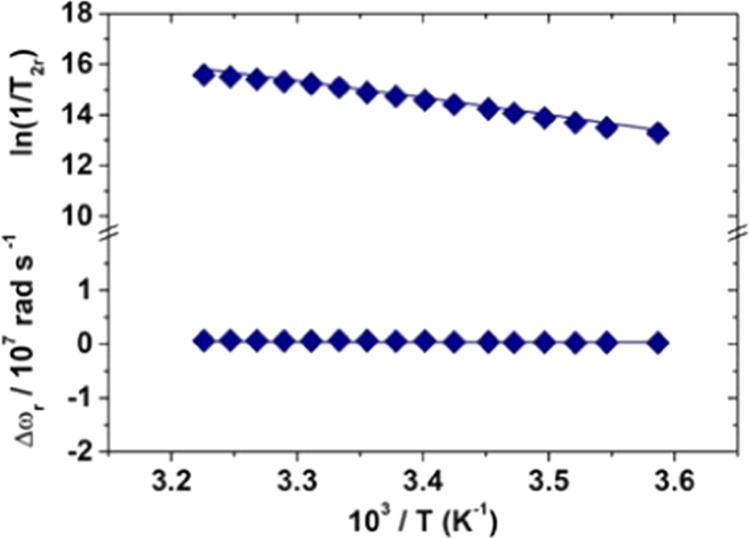
Reduced ^17^O NMR transverse relaxation rates and chemical shifts of [Fe(Tiron)_2_(H_2_O)_2_]^5–^ measured at 11.74 T ([Fe^3+^] = 7.94 mM, [Tiron] = 39.7 mM, pH = 4.34).

The presence of two metal-bound water molecules requires consideration of a strong IS contribution to relaxivity (eqs 3–5). Based on the literature data, we fixed the metal–proton distance of the coordinated water molecules (*r*_M–H_ = 2.70 Å). Information on the exchange dynamics of the two bound water molecules was obtained from recording and analyzing reduced ^17^O transverse relaxation rates (1/*T*_2r_) and chemical shifts (Δω_r_) data of an aqueous solution of the complex at 11.7 T (Figure 7). The transverse relaxation rates decrease as the temperature decreases, a behavior that indicates an intermediate/slow exchange regime, in which τ_M_ is not negligible compared to the transverse relaxation time of the two coordinated water molecules (τ_M_ ∼ *T*_2M_). Such a process is dominated by the scalar mechanism, which depends on the square of the hyperfine coupling constant *A*_O_/ℏ. The chemical shifts, Δω_r_, are directly proportional to *A*_O_/ℏ and therefore provide a complementary set of data. The ^17^O NMR data were analyzed using the Swift–Connick equations.^52^

We carried out a global fit of the ^1^H NMRD profiles and ^17^O NMR data, which is well known to be able to provide accurate and reliable values of the molecular parameters that influence relaxation. An excellent fit of the ^1^H NMRD and ^17^O NMR data was obtained with the parameters reported in Table 2. The analysis provided a value for τ_R_ (70 ps), which is entirely in line with those found for the corresponding complexes of Mn(II) or Gd(III) of similar molecular mass. The values of the electron relaxation parameters, Δ^2^ and τ_V_, fall within the range of values typical of the previously investigated Fe^3+^ complexes.^19^ The average residence lifetime of the coordinated water molecules, τ_M_ = 272 ns, is rather long compared to those found for [Fe(EDTA)(H_2_O)]^−^ (0.9 ns) and [Fe(CDTA)(H_2_O)]^−^ (36 ns).^19^ It is possible that in [Fe(Tiron)_2_(H_2_O)_2_]^5–^, where the metal ion is hexacoordinated, there is a lower degree of steric hindrance near the water coordination sites and therefore a stronger interaction with the Fe^3+^ ion. Finally, the parameters relating to the SS contribution are fully comparable with those calculated for [Fe(Tiron)_3_]^9–^, the only substantial difference being the number of second-sphere water molecules, i.e., two vs five. In summary, the relaxivity of [Fe(Tiron)_2_(H_2_O)_2_]^5–^ is dominated by the IS mechanism that provides a contribution of about 77% to *r*_1_, while the SS mechanism is only about 8% of the observed relaxivity (*r*_1_ = 6.5 mM^–1^ s^–1^; 3 T and 298 K). The latter, although rather small, is not a negligible contribution and without taking it into consideration, the best-fit procedure provides unsatisfactory results.

#### [Fe(Tiron)(H_2_O)_4_]^−^

**Figure 8:**
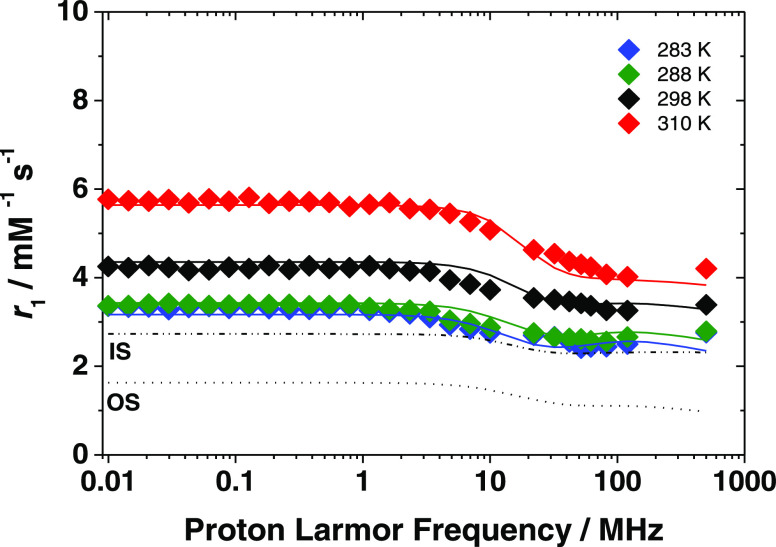
^1^H NMRD profiles (pH = 2.51) at different temperatures (283 (violet diamond solid), 288 (green diamond solid), 298 (black diamond solid), and 310 K (red diamond solid)) of [Fe(Tiron)(H_2_O)_4_]^−^ ([Fe^3+^] = 2.09 mM, [Tiron] = 5.22 mM). The solid lines correspond to the fit of the data. Dot–dashes and dots indicate respectively the inner and outer-sphere contribution to relaxivity at 298 K.

**Figure 9:**
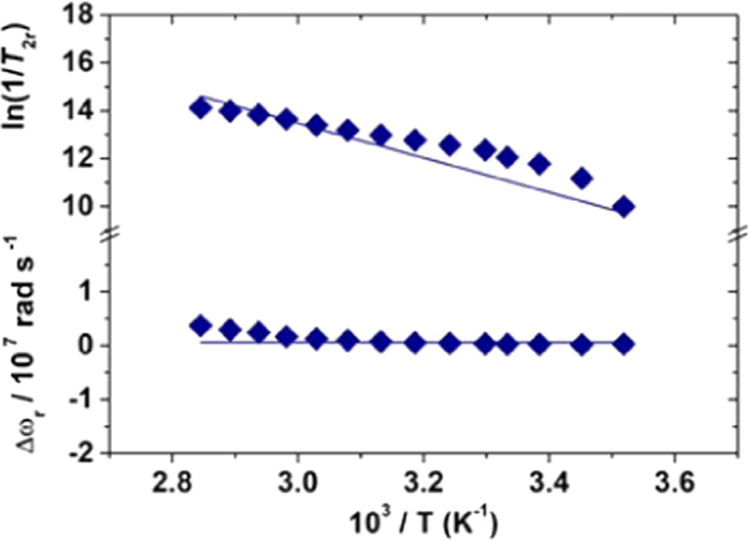
Reduced ^17^O NMR transverse relaxation rates and chemical shifts of [Fe(Tiron)(H_2_O)_4_]^−^ measured at 11.74 T ([Fe^3+^] = 8.25 mM, [Tiron] = 21.13 mM, pH = 2.17).

The presence of four coordinated water molecules in [Fe(Tiron)(H_2_O)_4_]^−^ (Figure S4) would suggest an IS contribution twice greater than that estimated for [Fe(Tiron)_2_(H_2_O)_2_]^5–^. This would imply a value of *r*_1_ of about 10 mM^–1^ s^–1^ at 3 T and 298 K. Instead, the measured value is only 3.3 mM^–1^ s^–1^ and the NMRD profiles, shown in Figure 8, clearly indicate a decrease in *r*_1_ across the entire range of frequencies. Furthermore, unlike [Fe(Tiron)_3_]^9–^ and [Fe(Tiron)_2_(H_2_O)_2_]^5–^, in this case, the relaxivity values decrease with decreasing temperature, a clear indication that the complex is in the slow exchange conditions (τ_M_ ≥ *T*_1M_), in which the long value of τ_M_ represents a limiting factor for *r*_1_. Following an approach completely similar to that used to study [Fe(Tiron)_2_(H_2_O)_2_]^5–^, we measured the ^17^O NMR values of *R*_2_ and paramagnetic shift as a function of temperature (Figure 9) and performed a simultaneous fitting procedure of the NMRD profiles and ^17^O data, obtaining the parameters reported in Table 2 that allow to reproduce the experimental values very well.

The average lifetime τ_M_ calculated is over 65 times longer than that found for [Fe(Tiron)_2_(H_2_O)_2_]^5–^ and of the same order of magnitude as that for the aqua ion, [Fe(H_2_O)_6_]^3+^.^19^ This is probably one of the longest reported values for an anionic metal complex of Gd^3+^, Mn^2+^, or Fe^3+^. Even the electron relaxation parameters, although falling within the range of characteristic values of Fe^3+^ complexes, are very similar to those reported for [Fe(H_2_O)_6_]^3+^.^19^ The hyperfine coupling constant *A*_O_/ℏ, on the other hand, has a value approximately intermediate between that of [Fe(CDTA)(H_2_O)]^−^ and that of [Fe(H_2_O)_6_]^3+^.^19^ The molecular correlation time τ_R_ is quite small, in excellent agreement with the reduced molecular mass of this complex. The results of the best-fit procedure are insensitive to the consideration of the presence of a contribution from SS. The presence of four water molecules in the inner coordination sphere of the metal ion makes the IS contribution largely dominant.

For all three complexes, we used the same value for the distance of closest approach of the OS water molecules. The value used is in line with previous studies on Fe^3+^ and other small complexes and it was fixed during the fit. Furthermore, it is worth noting that the fitting results are insensitive to variations of this parameter in the range of 3.4–3.6 Å (see Figures S5–S7).

The values of the bond distances obtained with DFT provide some hints on the trend observed for water exchange. For [Fe(H_2_O)_6_]^3+^, variable pressure ^17^O NMR measurements afforded an activation volume Δ*V*^‡^ = −5.4 cm^3^ mol^–1^,^53^ which points to an associative interchange mechanism. The introduction of Tiron ligands introduces some steric hindrance around the metal ion and makes the charge of the complex increasingly negative, an effect that is reflected in increased Fe–O_water_ and Fe–O_Tiron_ distances (Table 3). Thus, it is likely that the water exchange mechanism takes a more dissociative character as the negative charge of the complex increases. The faster water exchange in [Fe(Tiron)_2_(H_2_O)_2_]^5–^ is therefore probably the result of a favorable dissociative pathway facilitated by relatively weak Fe–O_water_ bonds. The absolute values of the hyperfine coupling constants *A*_O_/*ℏ*, calculated by DFT, decrease as the Fe–O_water_ distances increase, as would be expected. The trend predicted by DFT is in good agreement with the results obtained with ^17^O NMR experiments, which provides confidence on the results of the fits.

**Table 3:** Average Bond Distances, Hyperfine Coupling Constants, and Zero-Field Splitting Parameters Obtained with DFT and NEVPT2 Calculations

parameters	[Fe(Tiron)_3_]^9–^	[Fe(Tiron)_2_(H_2_O)_2_]^5–^	[Fe(Tiron)(H_2_O)_4_]^−^	[Fe(H_2_O)_6_]^3+^
Fe–O_water_ (Å)^a^		2.157	2.101	2.031
Fe–O_Tiron_ (Å)^a^	2.049	2.004	1.981	
*A*_O_/*ℏ* (10^6^ rad s^–1^)^a^		–71.0	–74.8	–99.3
*D* (cm^–1^)^b^	–0.074	–0.220	0.319	0.015
*E*/*D*^b^	0.101	0.144	0.094	0.008

a Values obtained with DFT.

b Data obtained with NEVPT2 calculations using CASSCF wave functions.

We have shown previously that electron relaxation affects the inner-sphere contribution to relaxivity at the imaging fields. Electron relaxation arises from the modulation of the zero-field splitting (ZFS) energy due to fluctuations of the metal coordination environment caused by vibrations and collisions with solvent molecules. Furthermore, a static ZFS mechanism was also shown to provide a significant contribution to electron relaxation. The ZFS lifts the degeneration of the magnetic sublevels of the *S* = 5/2 electronic ground state even in the absence of a magnetic field, generating three Kramers doublets, as discussed previously for both Mn^2+^ and Fe^3+^ complexes. Axial (*D*) and rhombic (*E*) ZFS parameters of the Tiron complexes investigated here were estimated using ab initio NEVPT2 calculations in an attempt to gain information of the factors that influence electron relaxation in Fe^3+^ complexes. The values of *D* calculated for [Fe(Tiron)_3_]^9–^ and [Fe(Tiron)_2_(H_2_O)_2_]^5–^ complexes are negative, a situation that indicates that two of the three Kramers doublets are higher in energy than the center of gravity. Conversely, the values of *D* are positive for [Fe(Tiron)(H_2_O)_4_]^−^ and [Fe(H_2_O)_6_]^3+^, where two Kramers doublets lie below the center of gravity (Figure S8). The absolute value of *D* follows the sequence [Fe(H_2_O)_6_]^3+^ < [Fe(Tiron)_3_]^9–^ < [Fe(Tiron)_2_(H_2_O)_2_]^5–^ < [Fe(Tiron)(H_2_O)_4_]^−^, which suggests that a more symmetrical coordination environment favors small ZFS, as observed previously for Gd(III) complexes. The [Fe(Tiron)_3_]^9–^ complex shows a lower value of |*D*| than [Fe(Tiron)_2_(H_2_O)_2_]^5–^, likely due to the more symmetrical coordination environment in the former. The change in the sign of *D* within this series of structurally related complexes is likely related to a progressive deviation of the twist angle of the parallel triangular faces of the coordination polyhedron from 60° in the octahedral [Fe(H_2_O)_6_]^3+^ complex to ∼42° in [Fe(Tiron)_3_]^9–^.

### Kinetic Studies

**Figure 10:**
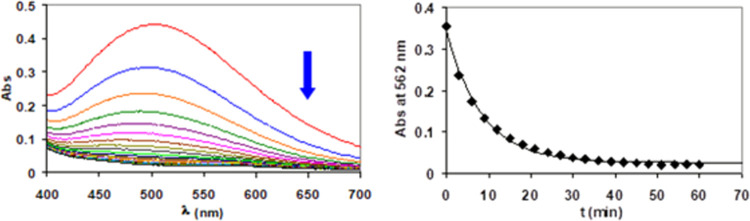
Absorption spectra and the absorbance values of the Fe(Tiron)*_x_*–CDTA reacting system at 562 nm ([Fe^3+^]*_t_* = 0.1 mM, [Tiron]*_t_* = 0.5 mM, [CDTA]*_t_* = 8 mM, pH = 6.50, 0.15 M NaNO_3_, 25 °C).

**Scheme 2:**
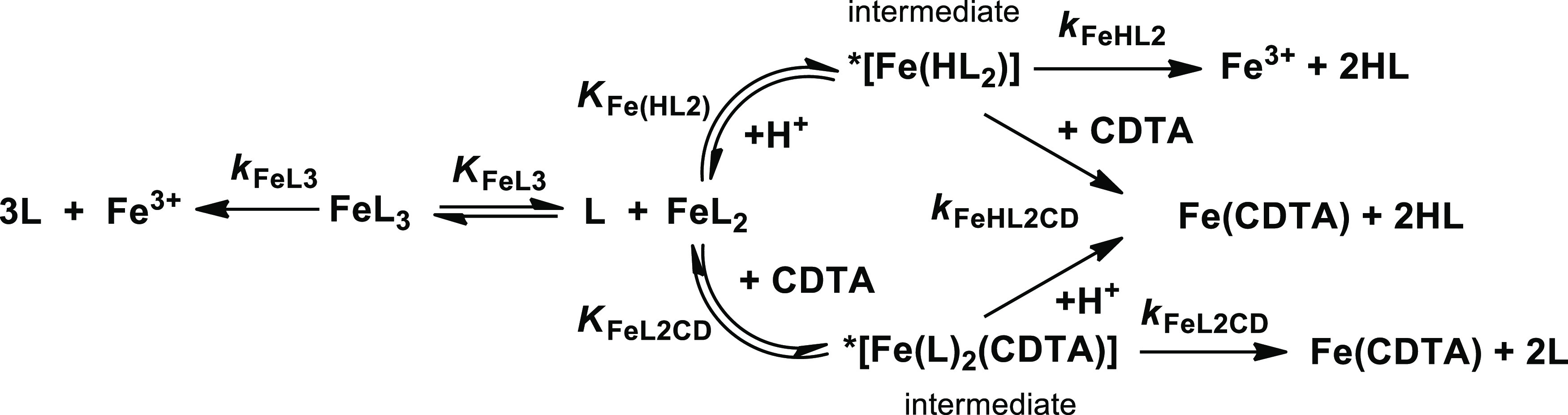
Proposed Mechanism for the Transchelation Reaction between Fe(Tiron)*_x_* and CDTA (L = Tiron, *x* = 2 and 3)

Together with thermodynamic stability data, a fundamental aspect concerning the properties of coordination compounds in solution is their kinetic inertness. The kinetic inertness of [Fe(Tiron)*_x_*]^(4*x*−3)–^ was determined by following the transchelation reactions between the Fe^3+^ complexes and the CDTA ligand via spectrophotometry, following the absorption band of the [Fe(Tiron)*_x_*]^(4*x*−3)–^ complexes (λ = 562 nm) in the pH range of 5.0–7.5. Based on the equilibrium data (Table 1), the [Fe(Tiron)_2_]^5–^ and [Fe(Tiron)_3_]^9–^ species dominate in the presence of a 5-fold Tiron excess in the pH range of 5.0–7.5 ([Fe^3+^] = 0.1 mM, [Tiron] = 0.5 mM, 0.15 M NaNO_3_, 25 °C) (Figure S9). Transchelation reactions of [Fe(Tiron)_2_]^5–^ and [Fe(Tiron)_3_]^9–^ species were investigated in the presence of 20–80 fold CDTA excess to guarantee pseudo-first-order kinetic conditions (eq 11, *x* = 2 and 3; *y* = 1 and 2; *z* = 1 and 2). Representative absorption spectra of [Fe(Tiron)*_x_*]^(4*x*−3)–^-CDTA reacting systems are shown in Figure 10. The proposed mechanism is shown in Scheme 2. The rate and equilibrium constants characterizing the transmetallation reactions of [Fe(Tiron)_2_]^5–^ and [Fe(Tiron)_3_]^9–^ complexes are summarized and compared with those of [Fe(EDTA)]^−^ and [Fe(CDTA)]^–^ complexes in Table 4. Definitions and equations used for the evaluation of the kinetic data are reported in the Supporting Information
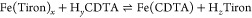
11

**Table 4:** Rate and Equilibrium Constants Characterizing the Transchelation Reactions of Fe(Tiron)*_x_*, Fe(EDTA)^−^, and Fe(CDTA)^−^ Complexes (*x* = 2 and 3, 0.15 M NaNO_3_, 25 °C)^a^

	Fe(Tiron)*_x_*	Fe(EDTA)^−^^19^	Fe(CDTA)^−^^19^
*k*_0_ (s^–1^)	(8 ± 1) × 10^–5^ (*k*_FeL_3__)	5 × 10^–6^	3.2 × 10^–7^
*k*_1_ (M^–1^ s^–1^)	(3.9 ± 0.5) × 10^3^		
*k*_4_ (M^–2^ s^–1^)	(7 ± 1) × 10^5^		
*k*_OH_ (M^–1^ s^–1^)		1.0	3.6 × 10^–3^
*k*_OH_^2^ (M^–2^ s^–1^)		1.4 × 10^3^	1.2
log* K*_FeL_3__	**9.20 (2)**		
log *K*_FeLH_–1__		**7.41**	**9.58**
*k*_d_ (s^–1^) at pH = 7.4	1.1 × 10^–4^	2.9 × 10^–6^	2.1 × 10^–9^
*t*_1/2_ (h) at pH = 7.4	1.8	66	8.9 × 10^4^

a Fe**L**_2_: *k*_1_ = *k*_FeHL_2__ × *K*_Fe(HL_2_)_; *k*_4_ = *k*_FeHL_2_CD_ × *K*_Fe(HL_2_)_ or *k*_FeHL_2_CD_ × *K*_FeL_2_CD_.

As it is shown in Figures S10 and S11, the *k*_d_ values increase with the increase of [H^+^] and [CDTA]*_t_* especially at pH < 6.0. The transchelation reaction of Fe(III) complexes takes place by the relatively slow dissociation of [Fe(Tiron)_2_]^5–^ and [Fe(Tiron)_3_]^9–^ species, which is followed by a fast reaction between the free Fe^3+^ ion and the exchanging CDTA ligand. The transchelation reaction can occur via the spontaneous dissociation of [Fe(Tiron)_3_]^9–^ (*k*_0_, eq S3), proton- (*k*_1_, eq S5), and CDTA-assisted dissociation (*k*_4_, eqs S8 and S9) of [Fe(Tiron)_2_]^5–^ through the formation of protonated *[FeHL_2_] (*K*_Fe(HL_2_)_, eq S4) or ternary *[FeL_2_(CDTA)] intermediates (*K*_FeL_2_CD_, eq S6), respectively. Interestingly, the stability constant of the [Fe(Tiron)_3_]^9–^ complex obtained by the kinetic studies (log *K*_FeL_3__ = 9.20, Table 4) agrees well with the log *K*_FeL_3__ value determined by pH-potentiometric and spectrophotometric studies (log *K*_FeL_3__ = 9.83(2), Table 3), which clearly confirms the correctness of the kinetic model used for the description of the [Fe(Tiron)*_x_*]^(4*x*−3)–^-CDTA reacting systems.

Since the transchelation reactions of [Fe(EDTA)]^−^ and [Fe(CDTA)]^−^ complexes with HBED take place by different pathways (the spontaneous (*k*_0_), first- (*k*_OH_) and second-order (*k*_OH_^2^) hydroxide-assisted dissociation),^19^ the dissociation rate constants (*k*_d_) and half-life (*t*_1/2_ = ln 2/*k*_d_) values of [Fe(Tiron)*_x_*]^(4*x*−3)–^, [Fe(EDTA)]^−^, and [Fe(CDTA)]^−^ complexes have been calculated near physiological conditions (Table 4, pH = 7.4 and 25 °C) to compare the kinetic inertness of the Fe^3+^ complexes (HBED: *N*,*N*-bis(2-hydroxybenzyl)ethylenediamine-*N*,*N*-diacetic acid). The dissociation rate constants (*k*_d_) of [Fe(EDTA)]^−^ and [Fe(CDTA)]^−^ complexes are about 37 and 50,000 times smaller than that of [Fe(Tiron)*_x_*]^(4*x*−3)–^, which indicates a relatively low inertness of [Fe(Tiron)*_x_*]^(4*x*−3)–^ species at pH = 7.4 due to the fast dissociation of [Fe(Tiron)_2_]^5–^. However, the relatively long dissociation half-life of the [Fe(Tiron)_3_]^9–^ species dominates at pH ≥ 8.0 (*t*_1/2_ = 4.1 h, 25 °C). Thus, the very slow decoordination of the first Tiron ligand in [Fe(Tiron)_3_]^9–^ results in the higher kinetic inertness of the [Fe(Tiron)_3_]^9–^ species than that of [Fe(Tiron)_2_]^5–^.

## Conclusions

The bidentate ligand Tiron finds common use in chemical research and various industrial applications. In analogy with numerous other mono- and bidentate ligands, Tiron can coordinate with Fe^3+^ in aqueous solution in a variety of ways, giving rise to metal complexes that differ in stoichiometry, hydration state, and overall electric charge. These species have a range of existence strictly controlled by the pH of the solution and dependent on the ligand-to-metal-ion molar ratio. The detailed characterization of these species is a difficult challenge and an open problem. This work has shown that the combined use of potentiometric data, ^1^H and ^17^O NMR relaxometric studies, and theoretical calculations represents a very effective approach to obtain relevant structural and dynamic information on each of the species of the Fe-Tiron system.

Equilibrium data obtained by the combination of pH potentiometry and vis spectrophotometry allows the accurate determination of the stability and protonation constants of [Fe(Tiron)]^−^, [Fe(Tiron)_2_]^5–^, [Fe(Tiron)_3_]^9–^, and [Fe(Tiron)_2_H_–1_]^6–^ species formed in Fe(III)-Tiron systems. In the presence of 4-fold Tiron excess, there is a stepwise formation of [Fe(Tiron)_2_]^5–^, [Fe(Tiron)_3_]^9–^, and [Fe(Tiron)_2_H_–1_]^6–^ species in the −log[H^+^] ranges of 0.0–3.0, 3.0–6.0, and 6.0–8.0, respectively. However, the presence of lower Tiron excess ([Tiron]/[Fe^3+^] = 2.5 and 3.0) results in the formation of a [Fe(Tiron)_2_H_–1_]^6–^ species at −log[H^+^] ≥ 6.0. The kinetic parameters characterizing the transmetallation reactions of [Fe(Tiron)_2_]^5–^ and [Fe(Tiron)_3_]^9–^ with CDTA reveal that the dissociation rate of [Fe(Tiron)_3_]^9–^ is relatively slow (*t*_1/2_ = 4.1 h, 25 °C) due to the slow decoordination of the first Tiron ligand. The multinuclear and multifrequency NMR relaxometric data provide a set of consistent and sufficiently accurate molecular parameters that well-describe the structure, hydration state, molecular tumbling motion, and dynamics of the solvent exchange process of the species present in different pH ranges of the Fe^3+^/Tiron system. This information is very useful and extremely hard to obtain through other experimental procedures. Theoretical calculations are of considerable help in guiding the analysis of the relaxometric data and the correct interpretation of the obtained molecular parameters. In particular, DFT calculations provide information on the number of second-sphere water molecules and their distance to the paramagnetic center, as well as the ^17^O hyperfine coupling constants responsible for the scalar contribution to the NMR transverse relaxation rates and chemical shifts. Furthermore, CASSCF/NEVPT2 calculations provide information on electronic relaxation, which is still poorly understood. The results reported here suggest that the symmetry of the metal coordination environment plays a significant role in electron relaxation.

In conclusion, the results presented here represent a step forward toward the development of an effective methodology to understand the behavior in aqueous media of paramagnetic species characterized by a complex pH-dependent speciation.

## Supporting Information Available

Details on the kinetic studies of the transchelation reactions between [Fe(Tiron)*_x_*]^(4*x*−3)^ complexes (*x* = 2 and 3) and CDTA; calculated NMRD profiles; and DFT and NEVPT2 calculations (PDF)
