# Supplementary material for: Characterization of the Fe(III)-Tiron System in Solution through an Integrated Approach Combining NMR Relaxometric, Thermodynamic, Kinetic, and Computational Data
Source: Inorg Chem. 2023 Mar 2;62(10):4272–83. doi: 10.1021/acs.inorgchem.2c04393 (PMC10015466; doi:10.1021/acs.inorgchem.2c04393)
Supplement: Supplementary file 1 — ic2c04393_si_001.pdf [file ic2c04393_si_001.pdf]

Electronic Supporting Information (ESI)

Characterization of the Fe(III)-*Tiron* system in solution through an integrated approach combining NMR relaxometric, thermodynamic, kinetic and computational data

Alessandro Nucera,[†] *Fabio Carniato,*[†‡] *Zsolt Baranyai,*[#*] *Carlos Platas-Iglesias,*[§*] *Mauro Botta.*[†‡*]

† Dipartimento di Scienze e Innovazione Tecnologica, Università del Piemonte Orientale, Viale Teresa Michel 11, 15121 Alessandria (Italy). mauro.botta@uniupo.it

‡ Magnetic Resonance Platform (PRISMA-UPO), Università del Piemonte Orientale, Viale Teresa Michel 11, 15121 Alessandria (Italy).

[#] Bracco Research Centre, Bracco Imaging S.p.A., Via Ribes 5, 10010, Colleretto Giacosa, Italy. zsolt.baranyai@bracco.com

[§] Departamento de Química Fundamental, Facultade de Ciencias, Universidade da Coruña, Campus da Zapateira-Rúa da Fraga 10, 15008 A Coruña, Spain. carlos.platas.iglesias@udc.es

Contents

Figure S1. Species distribution of Fe(III)–Tiron system ([Fe^{3+}]=1.88 mM, [L = *Tiron*]=94.0 mM, 0.15 M $NaNO_3$, 25°C)........3

Figure S2. Structure of the [Fe(*Tiron*)$_3$]$^{9-}$·5H_2O system optimized at the uTPSSh/Def2-TZVPP level. Bond distances (Å): Fe(1)-O(1) 2.091; Fe(1)-O(2) 2.014; Fe(1)-O(3) 2.026; Fe(1)-O(4) 2.072; Fe(1)-O(5) 2.059; Fe(1)-O(6) 2.033. 4

Figure S3. Structure of the [Fe(*Tiron*)$_2$(H_2O)$_2$]$^{5-}$·9H_2O system optimized at the uTPSSh/Def2-TZVPP level. Bond distances (Å): Fe(1)-O(1) 2.046; Fe(1)-O(2) 1.966; Fe(1)-O(3) 2.011; Fe(1)-O(4) 1.993; Fe(1)-O(5) 2.152; Fe(1)-O(6) 2.161. 5

Figure S4. Structure of the [Fe(*Tiron*)(H_2O)$_4$]$^{-}$·13H_2O system optimized at the uTPSSh/Def2-TZVPP level. Bond distances (Å): Fe(1)-O(1) 2.964; Fe(1)-O(2) 1.997; Fe(1)-O(3) 2.101; Fe(1)-O(4) 2.070; Fe(1)-O(5) 2.120; Fe(1)-O(6) 2.111. 6

Figure S5. Calculated NMRD profiles of [Fe(*Tiron*)$_3$]$^{9-}$ by varying the *a* parameter from 3.4 (left) to 3.6 (right) Å........7

Figure S6. Calculated NMRD profiles of [Fe(*Tiron*)$_2$(H_2O)$_2$]$^{5-}$ by varying the *a* parameter from 3.4 (left) to 3.6 (right) Å........7

Figure S7. Calculated NMRD profiles of [Fe(*Tiron*)(H_2O)$_4$]$^{-}$ by varying the *a* parameter from 3.4 (left) to 3.6 (right) Å........7

Figure S8. Splitting of the Kramers doublets obtained with CASSCF/NEVPT2 calculations for Fe(III) complexes and the corresponding signs of *D*. 8

Kinetic inertness of [Fe(*Tiron*)$_x$]$^{(4x-3)-}$ complexes (x=2 and 3) 9

Figure S9. Species distribution of Fe(III)–*Tiron* system ([Fe^{3+}]=0.1 mM, [L = *Tiron*]=0.5 mM, 0.15 M $NaNO_3$, 25°C)........9

Figure S10. Pseudo-first order rate constants (k_d) characterize the transchelation reactions of [Fe(*Tiron*)$_x$]$^{(4x-3)-}$ with CDTA ligand as a function of pH (**A**) and [H^+] (**B**) (x=2 and 3, ([Fe^{3+}]$_t$=0.1 mM, [*Tiron*]$_t$=0.5 mM, [CDTA]$_t$=2.0 (◆), 4.0 (■), 6.0 (▲) and 8.0 mM (●), pH=6.50, 0.15 M $NaNO_3$, 25°C). 10

Figure S11. Pseudo-first order rate constants (k_d) characterize the transchelation reactions of [Fe(*Tiron*)$_x$]$^{(4x-3)-}$ with CDTA ligand as a function of [CDTA]$_t$. (x=2 and 3, ([Fe^{3+}]$_t$=0.1 mM, [*Tiron*]$_t$=0.5 mM, pH=5.04 (◆), 5.56 (■), 6.16 (▲), 6.74 (●) and 7.44 (✳) pH=6.50, 0.15 M $NaNO_3$, 25°C)........10

Table S1. Optimized Cartesian coordinates (Å) of the [Fe(*Tiron*)$_3$]$^{9-}$·5H_2O system obtained with DFT calculations (0 Imaginary Frequencies).

Table S2. Optimized Cartesian coordinates (Å) of the [Fe(*Tiron*)$_2$(H_2O)$_2$]$^{5-}$·9H_2O system obtained with DFT calculations (0 Imaginary Frequencies). 15

Table S3. Optimized Cartesian coordinates (Å) of the [Fe(*Tiron*)(H_2O)$_4$]$^{-}$·13H_2O system obtained with DFT calculations (0 Imaginary Frequencies). 17

References 18

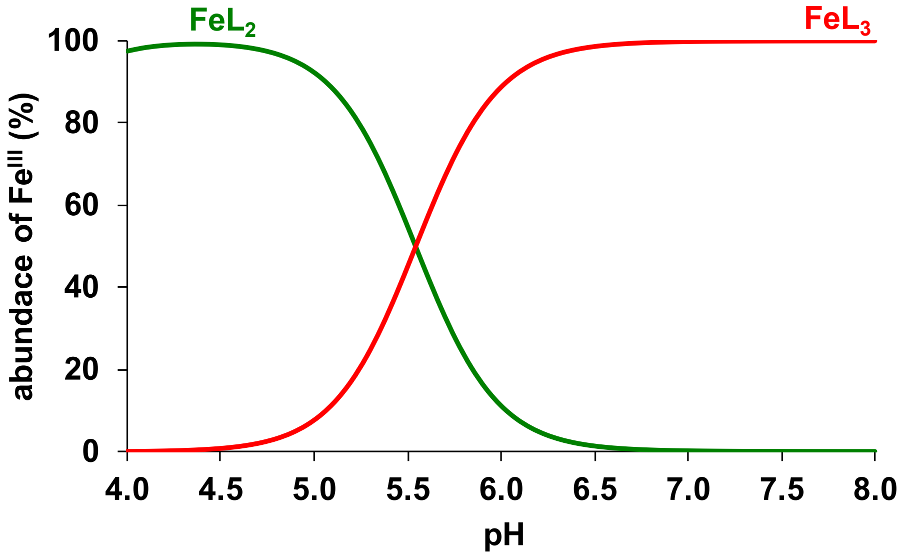

Figure S1. Species distribution of Fe(III)–*Tiron* system ([Fe^{3+}]=1.88 mM, [L = *Tiron*]=94.0 mM, 0.15 M $NaNO_3$, 25°C)

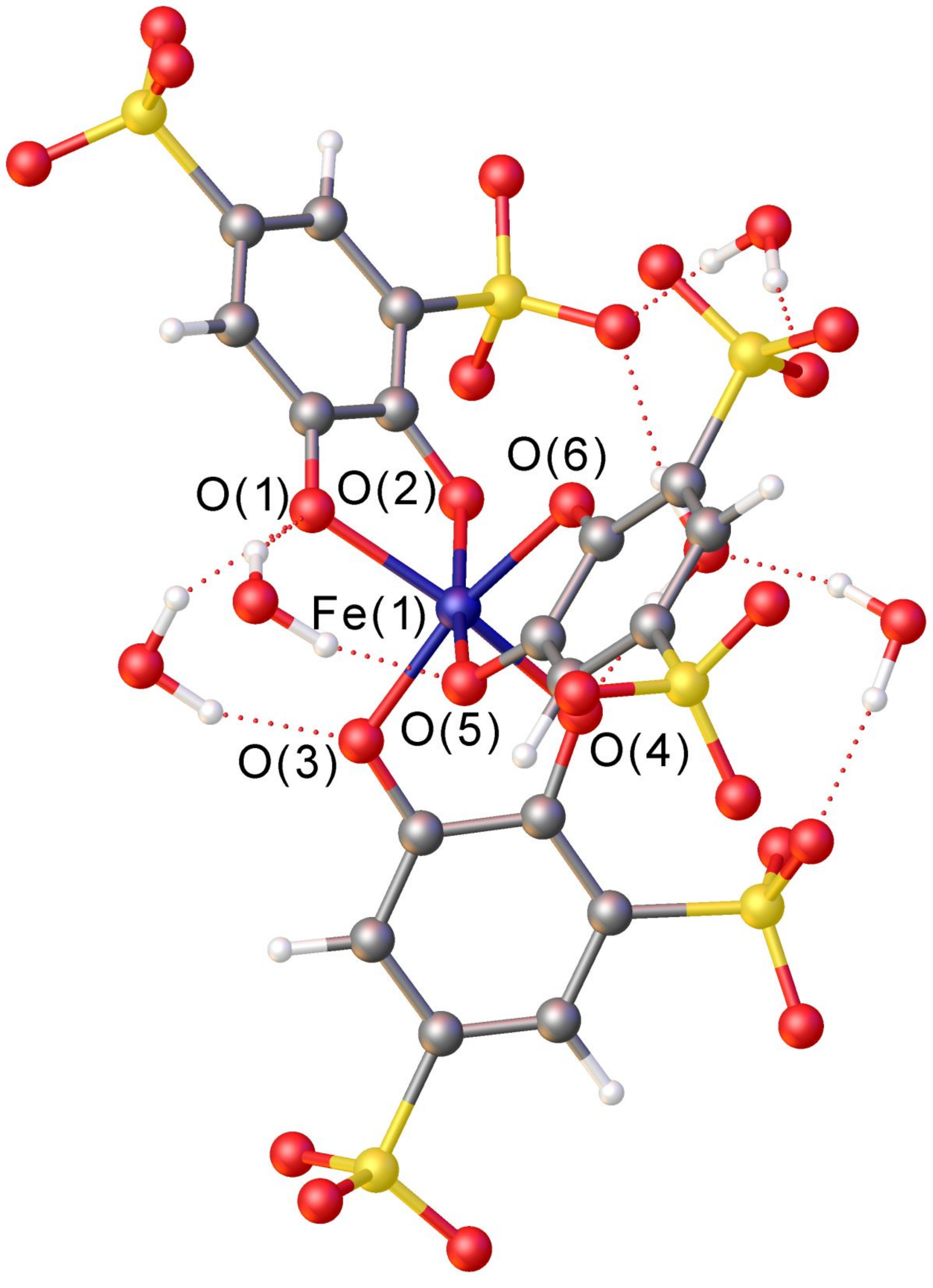

Figure S2. Structure of the [Fe(*Tiron*)$_3$]$^{9-}$·5H_2O system optimized at the uTPSSh/Def2-TZVPP level. Bond distances (Å): Fe(1)-O(1) 2.091; Fe(1)-O(2) 2.014; Fe(1)-O(3) 2.026; Fe(1)-O(4) 2.072; Fe(1)-O(5) 2.059; Fe(1)-O(6) 2.033.

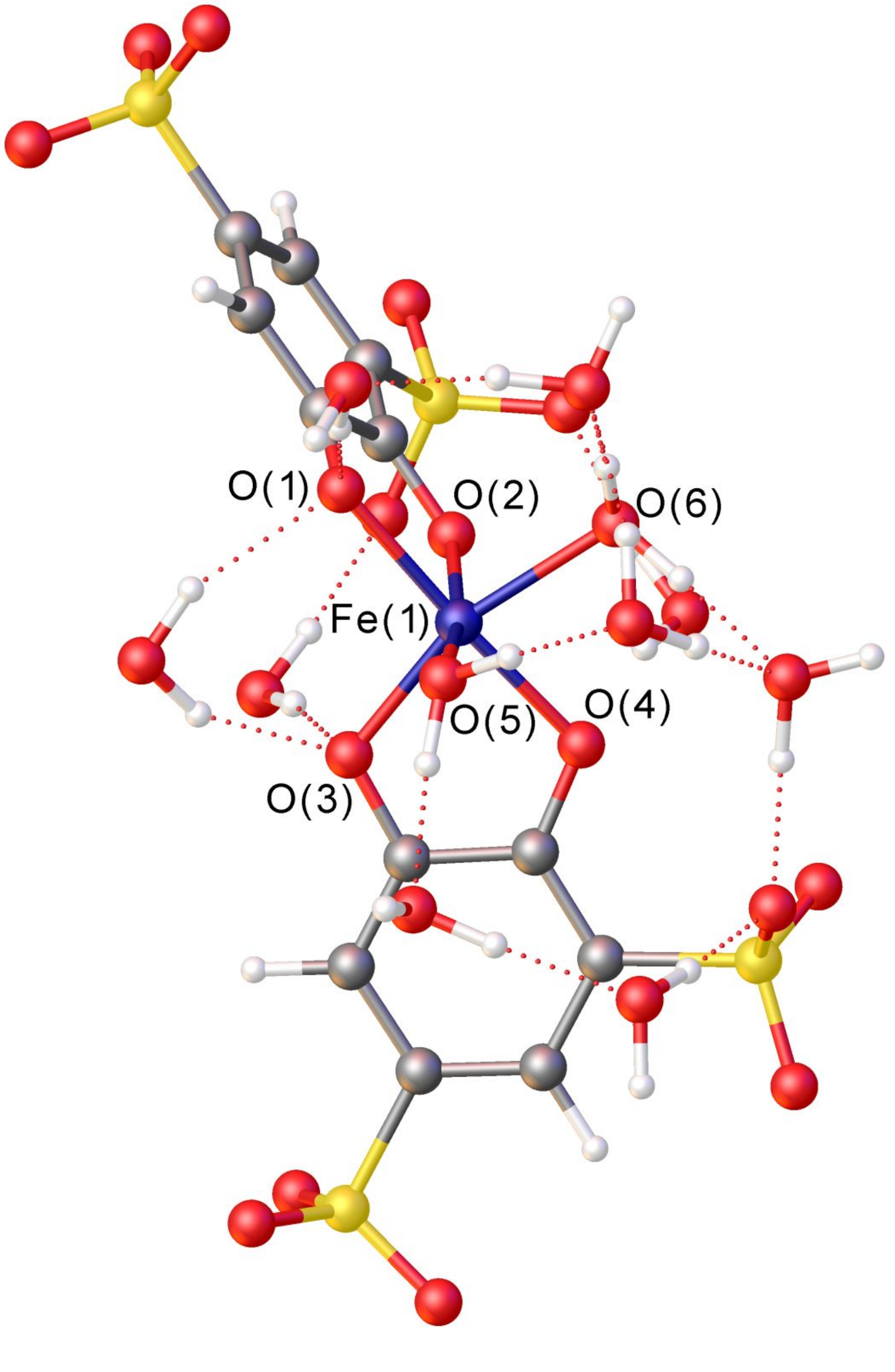

Figure S3. Structure of the $[Fe(\textit{Tiron})_2(H_2O)_2]^{5-}\cdot 9H_2O$ system optimized at the uTPSSh/Def2-TZVPP level. Bond distances (Å): Fe(1)-O(1) 2.046; Fe(1)-O(2) 1.966; Fe(1)-O(3) 2.011; Fe(1)-O(4) 1.993; Fe(1)-O(5) 2.152; Fe(1)-O(6) 2.161.

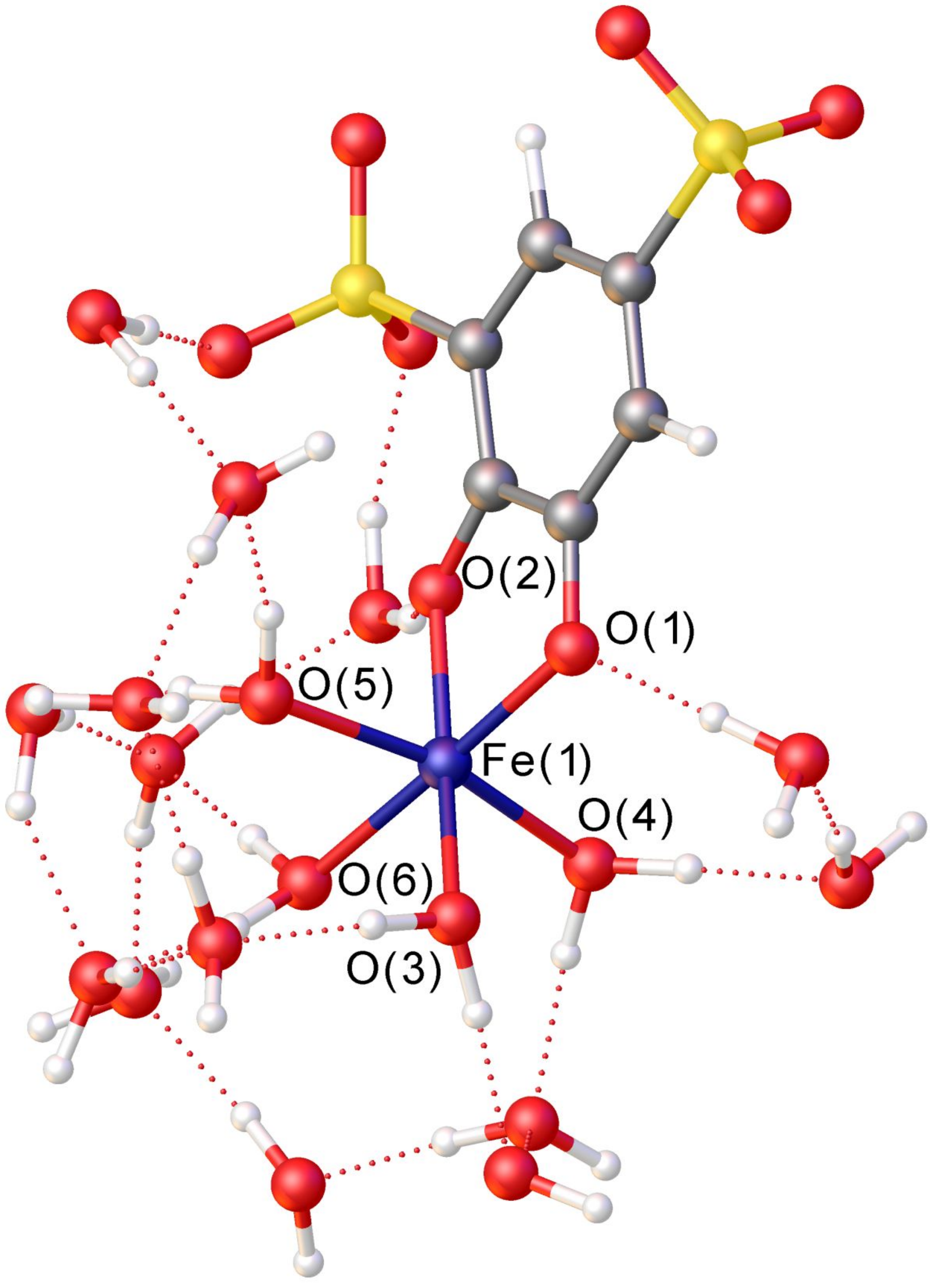

Figure S4. Structure of the [Fe(*Tiron*)$(H_2O)_4]^{-}\cdot 13H_2O$ system optimized at the uTPSSh/Def2-TZVPP level. Bond distances (Å): Fe(1)-O(1) 2.964; Fe(1)-O(2) 1.997; Fe(1)-O(3) 2.101; Fe(1)-O(4) 2.070; Fe(1)-O(5) 2.120; Fe(1)-O(6) 2.111.

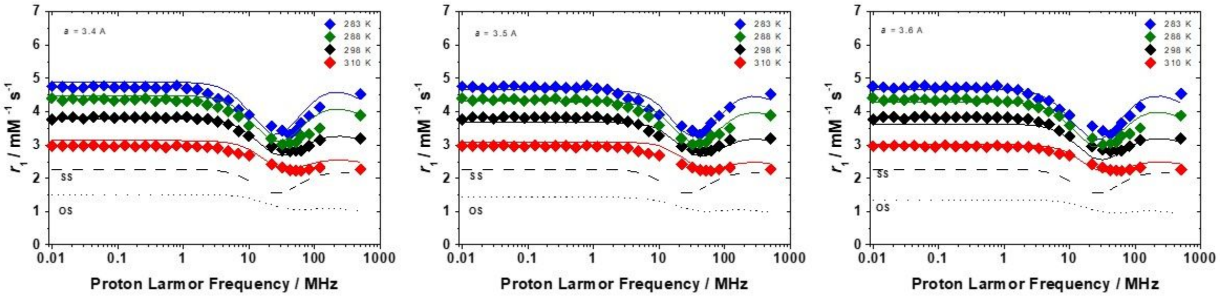

Figure S5. Calculated NMRD profiles of $[Fe(\textit{Tiron})_3]^{9-}$ by varying the *a* parameter from 3.4 (left) to 3.6 (right) Å.

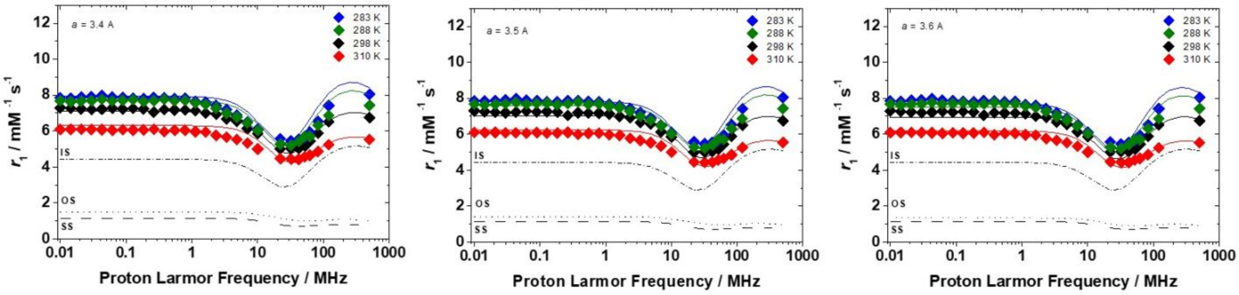

Figure S6. Calculated NMRD profiles of $[Fe(\textit{Tiron})_2(H_2O)_2]^{5-}$ by varying *a* from 3.4 (left) to 3.6 (right) Å.

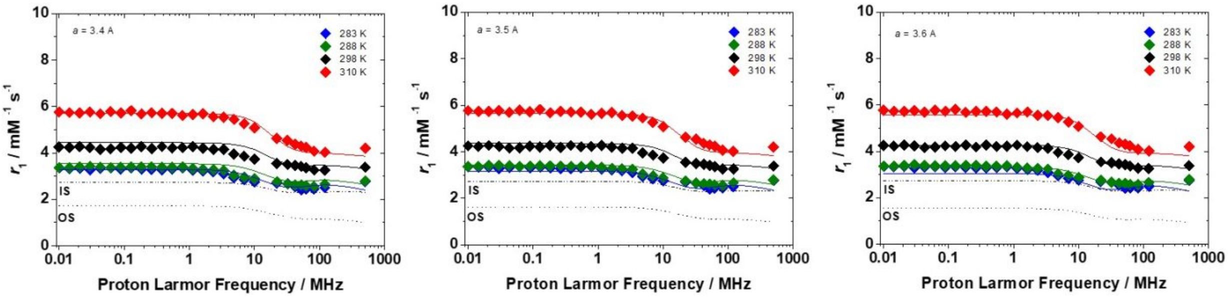

Figure S7. Calculated NMRD profiles of $[Fe(\textit{Tiron})(H_2O)_4]^{-}$ by varying *a* from 3.4 (left) to 3.6 (right) Å.

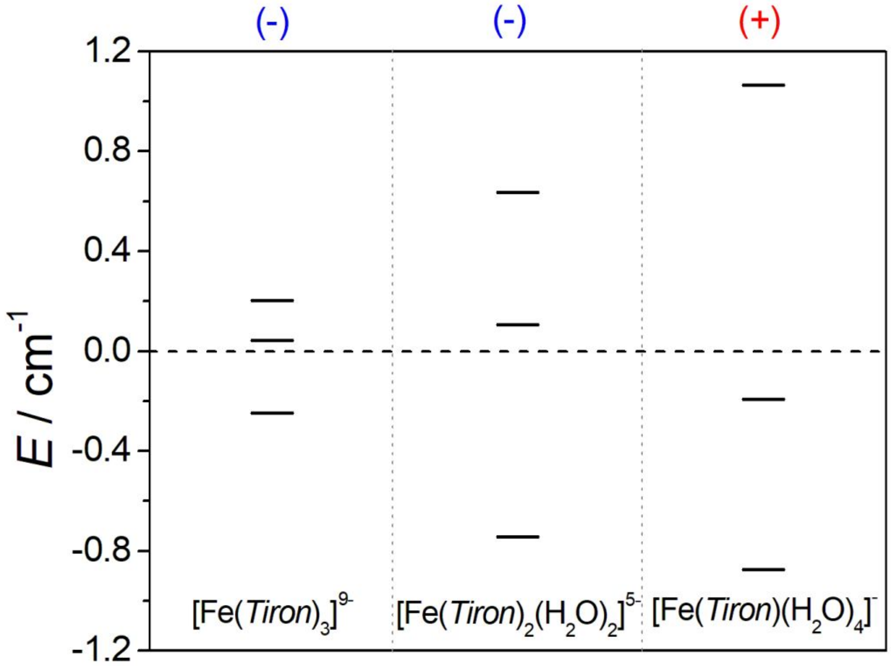

Figure S8. Splitting of the Kramers doublets obtained with CASSCF/NEVPT2 calculations for Fe(III) complexes and the corresponding signs of *D*.

Kinetic inertness of $[Fe(\textit{Tiron})_x]^{(4x-3)-}$ complexes (x=2 and 3)

The kinetic inertness of $[Fe(\textit{Tiron})_x]^{(4x-3)-}$ (x=2 and 3) was determined by following the transmetallation reaction between the Fe^{III}-complexes and CDTA ligand (Eq. S1) with spectrophotometry on the absorption band of the $Fe(\textit{Tiron})_x$-complexes in the presence of CDTA excess in the pH rang e 5.0 – 7.5. Species distribution of Fe(III)–*Tiron* system in the condition of the kinetic studies is shown in Figure S1 ($[Fe^{3+}]$=0.1 mM, [L = *Tiron*]=0.5 mM, 0.15 M $NaNO_3$, 25°C).

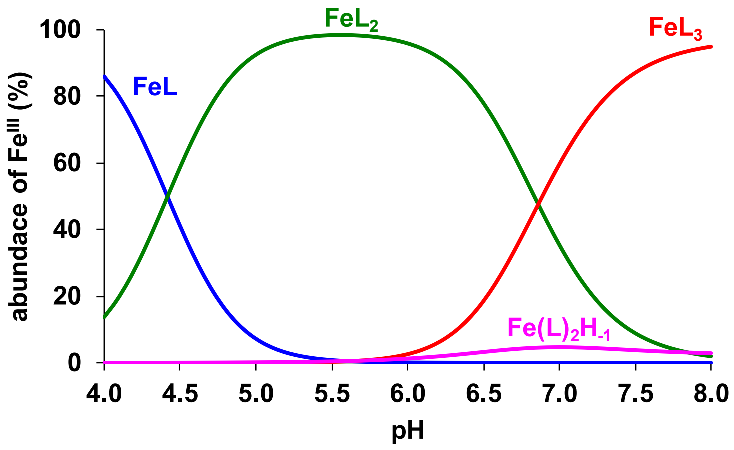

Figure S9. Species distribution of Fe(III)–*Tiron* system ($[Fe^{3+}]$=0.1 mM, [L = *Tiron*]=0.5 mM, 0.15 M $NaNO_3$, 25°C).

$$[Fe(\textit{Tiron})_x]^{(4x-3)-} + H_yCDTA \rightleftharpoons [Fe(CDTA)]^- + H_z\,\textit{Tiron} \qquad (S1)$$

where x=2 and 3; y=1 and 2; z=1 and 2. In the presence of excess of the exchanging CDTA ligand, the transchelation can be treated as a pseudo-first-order process and the reaction rates can be expressed with the Eq. (S2), where k_d is a pseudo-first-order rate constant and $[FeL]_{tot}$ is the total concentration of the $[Fe(\textit{Tiron})_x]^{(4x-3)-}$ complex.

$$-\frac{d[FeL]_{tot}}{dt} = k_d[FeL]_{tot} \qquad (S2)$$

The rates of the transchelation reactions were studied at different concentrations of the exchanging CDTA ligand in the pH range 5.0 – 7.5. The obtained rate constants k_d characterizing the transchelation reactions of $[Fe(Tiron)_x]^{(4x-3)-}$ with CDTA are presented as a function of $[H^+]$, pH and $[CDTA]_{tot}$ in Figures S10 and S11.

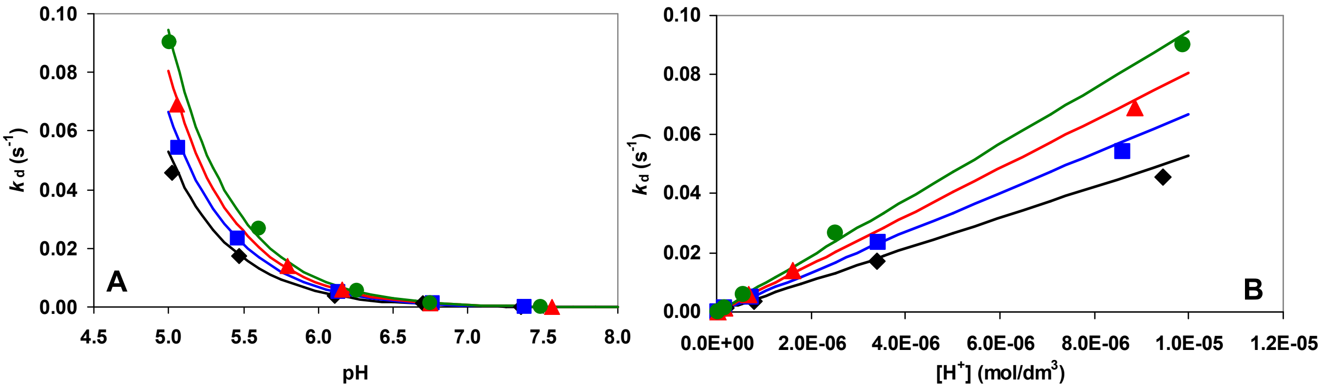

Figure S10. Pseudo-first order rate constants (k_d) characterize the transchelation reactions of $[Fe(Tiron)_x]^{(4x-3)-}$ with CDTA ligand as a function of pH (**A**) and $[H^+]$ (**B**) (x=2 and 3, ($[Fe^{3+}]_t$=0.1 mM, $[Tiron]_t$=0.5 mM, $[CDTA]_t$=2.0 (◆), 4.0 (■), 6.0 (▲) and 8.0 mM (●), pH=6.50, 0.15 M $NaNO_3$, 25°C).

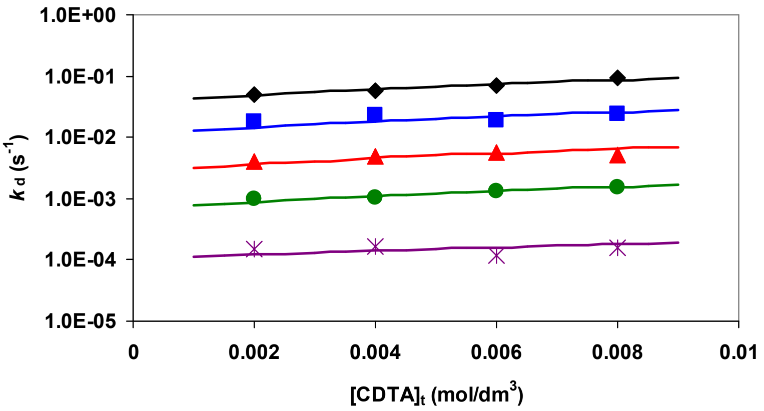

Figure S11. Pseudo-first order rate constants (k_d) characterize the transchelation reactions of $[Fe(Tiron)_x]^{(4x-3)-}$ with CDTA ligand as a function of $[CDTA]_t$. (x=2 and 3, ($[Fe^{3+}]_t$=0.1 mM, $[Tiron]_t$=0.5 mM, pH=5.04 (◆), 5.56 (■), 6.16 (▲), 6.74 (●) and 7.44 (✳) pH=6.50, 0.15 M $NaNO_3$, 25°C).

The k_d values increase with increase of $[H^+]$ and $[CDTA]_t$ especially at pH < 6.0. By taking into account the species distribution of the Fe(III)–*Tiron* systems in the presence of *Tiron* excess ($[Tiron]/[Fe^{3+}] \geq 5.0$), the transchelation reaction of Fe(III)-complexes takes place by the relatively slow dissociation of $[Fe(Tiron)_2]^{5-}$ (FeL_2) and $[Fe(Tiron)_3]^{9-}$ (FeL_3) species at pH<6.0 and pH>6.0, which is followed by a fast reaction between the free Fe^{3+}-ion and the exchanging CDTA ligand. The k_d vs. $[H^+]$ data can be expressed as a first-order function of $[H^+]$, which might be interpreted by the spontaneous dissociation of the $[Fe(Tiron)_3]^{9-}$ species (Eq. (S3)) and the proton assisted dissociation of the $[Fe(Tiron)_2]^{5-}$ (Eq. (S5)) dominated at pH>6.0 and pH<6.0, respectively. The proton assisted dissociation of the complex can occur by the equilibrium formation of the protonated $[FeH(Tiron)_2]^{4-}$ ($FeHL_2$) species (Eq. (S4)).

$$FeL_3 \xrightarrow{k_{FeL3}} Fe^{3+} + 3L \qquad \textit{(S3)}$$

$$FeL_2 + H^+ \rightleftharpoons FeHL_2 \qquad \textit{(S4)}$$

$$K_{FeHL_2} = \frac{[FeHL_2]}{[FeL_2][H^+]}$$

$$FeHL_2 \xrightarrow{k_{FeHL2}} Fe^{3+} + 2H_xL \qquad \textit{(S5)}$$

The transchelation reaction can also take place with the direct attack of the exchanging CDTA ligand on the $[Fe(Tiron)_2]^{5-}$, via the formation of ternary $*[Fe(Tiron)_2(CDTA)]^{9-}$ ($FeL_2(CDTA)$) intermediate (Eq. (S6)). The formation of the $*[Fe(Tiron)_2(CDTA)]^{9-}$ intermediate might takes place by the substitution of the two inner-sphere H_2O water molecules by two donor atoms of the CDTA ligand. It can be assumed that in the ternary $*[Fe(Tiron)_2(CDTA)]^{9-}$ intermediate the functional groups of the *Tiron* ligand are slowly substituted by the CDTA ligand by the formation of the $Fe(CDTA)^-$ complex (Eq. (S7)).

K_{FeL2CD}

$$FeL_2 + CDTA \rightleftharpoons FeL_2(CDTA) \quad (S6)$$

$$K_{FeL_2CD} = \frac{[FeL_2(CDTA)]}{[FeL_2][CDTA]_t}$$

$$FeL_2(CDTA) \xrightarrow{k_{FeL2CD}} Fe(CDTA)^- + 2L \quad (S7)$$

The k_d *vs*. $[H^+]$ and $[CDTA]_t$ graphs in Figures S10 and S11 reveal that the increase of $[CDTA]_t$ results in the increase in the k_d values at lower pH values. This phenomenon can be interpreted by the H^+ assisted dissociation of the ternary $*[Fe(Tiron)_2(CDTA)]^{9-}$ intermediate (Eq. (S8)) or by the CDTA assisted dissociation of the protonated $[FeH(Tiron)_2]^{4-}$ intermediate (Eq. (S9)) formed in the presence of the larger concentration of the H^+ and in the presence of the high excess of the exchanging CDTA ligand, respectively. Since the two processes cannot be discriminated, the contribution of these pathways to the transchelation of the Fe(III)-complex can be considered as a simultaneous proton and CDTA assisted dissociation of the $[Fe(Tiron)_2]^{5-}$ species. Similar phenomena were identified in the kinetic studies of the transmetallation reaction of [Gd(DTPA-BMA)] and $[Gd(DTPA)]^{2-}$ complexes in the presence of protonated citrate.[1,2]

$$FeL_2(CDTA) + H^+ \xrightarrow{k_{FeHL2CD}} Fe(CDTA)^- + 2HL \quad (S8)$$

$$FeHL_2 + CDTA \xrightarrow{k_{FeHL2CD}} Fe(CDTA)^- + 2HL \quad (S9)$$

By taking into account all possible pathways, the rate of the transmetallation of $[Fe(Tiron)_x]^{(4x-3)-}$ can be expressed by Eq. (S10) or Eq. (S11), where the $[FeHL_2]$, $[FeL_2(CDTA)]$ and $[FeL_3]$ are the concentrations of the protonated, ternary and tertiary Fe^{III}-complexes, respectively:

$$-\frac{[FeL]_{tot}}{dt} = k_{FeL_3}[FeL_3] + k_{FeHL_2}[FeHL_2] + k_{FeL_2CD}[FeL_2(CDTA)] + k_{FeHL_2CD}[FeL_2(CDTA)][H^+] \quad (S10)$$

$$-\frac{[FeL]_{tot}}{dt} = k_{FeL_3}[FeL_3] + k_{FeHL_2}[FeHL_2] + k_{FeL_2CD}[FeL_2(CDTA)] + k_{FeHL_2CD}[FeHL_2][CDTA] \quad (S11)$$

If we take into account the total concentration of the Fe^{III}-complexes ($[FeL]_{tot}=[FeL_2]+[FeL_3]+[FeHL_2]+[FeL_2(CDTA)]$), the concentration of the *Tiron* excess ($[L]_{tot} - [FeL_2]=[L]+[HL]+[H_2L]$), the stability constant of the $[Fe(\mathit{Tiron})_3]^{9-}$ species ($K_{FeL3}=[FeL_3]/[FeL_2][L]$), the protonation constant of the FeL_2 species (Eq. (S4)), the stability constant of the ternary $^*[FeL_2(CDTA)]$ complex (Eq. (S6)) and Eq. (S2), the pseudo-first-order rate constant can be expressed as follows:

$$k_d = \frac{k_0[L] + k_1[H^+] + k_3[CDTA] + k_4[H^+][CDTA]}{1 + K_{FeL_3}[L] + K_{FeHL_2}[H^+] + K_{FeL_2CD}[CDTA]} \quad (S12)$$

where $k_0=k_{FeL3}\times K_{FeL3}$, $k_1=k_{FeHL2}\times K_{Fe(HL2)}$, $k_3=k_{FeL2CD}\times K_{FeL2CD}$ and $k_4=k_{FeHL2CD}\times K_{FeL2CD}$ or $k_4=k_{FeHL2CD}\times K_{FeHL2}$ and $[L]=([L]_{tot}-[FeL_2])/(1+K_1^H[H^+]+K_1^HK_2^H[H^+]^2)$ (K_1^H and K_2^H are the protonation constants of the *Tiron* ligand). The rate constants k_0 represents the spontaneous dissociation of the $[Fe(\mathit{Tiron})_3]^{9-}$ complex, k_1, k_3 and k_4 are characteristic of the proton-, CDTA, proton and CDTA assisted dissociation of the $[Fe(\mathit{Tiron})_2]^{5-}$, respectively. By taking into account the low protonation constant of the $[Fe(\mathit{Tiron})_2]^{5-}$ species ($K_{FeHL2}<100$) and the relatively low stability constant of the ternary $^*[Fe(\mathit{Tiron})_2(CDTA)]^{9-}$ species ($K_{FeL2CD}<100$) as it shown in Figure S7, the denominator of Eq. (S12) ($1 >> K_{FeHL2}[H^+]+K_{FeL2CD}[CDTA]$) can be neglected, so Eq. (S12) can be simplified in the form of Eq. (S13).

$$k_d = \frac{k_0[L] + k_1[H^+] + k_3[CDTA] + k_4[H^+][CDTA]}{1 + K_{FeL_3}[L]} \quad (S13)$$

The rate, protonation and stability constants characterize the transchelation of $[Fe(\mathit{Tiron})_x]^{(4x-3)-}$ were calculated by fitting the k_d values in Figures S10 and S11 to the Eq. (S13). However, the k_3 values obtained are very low and the error in them is very high, indicating the

unimportance of the spontaneous dissociation of the ternary *[Fe(*Tiron*)$_2$(CDTA)]$^{9-}$ intermediate (Eq. (S7)).

Table S1. Optimized Cartesian coordinates (Å) of the [Fe(*Tiron*)$_3$]$^{9-}$·5H_2O system obtained with DFT calculations (0 Imaginary Frequencies).

Center Number	Atomic Number		Coordinates (Angstroms) X	Y	Z
1	8	0	1.481831	-0.792879	-1.925526
2	8	0	-1.210854	-1.853016	-1.426484
3	8	0	1.270732	-1.557012	0.556187
4	8	0	0.742713	1.212782	0.307938
5	26	0	-0.036631	-0.470331	-0.524410
6	6	0	2.600115	-1.216727	-1.339509
7	6	0	2.472247	-1.616519	0.036334
8	6	0	3.831208	-1.306675	-1.977766
9	6	0	3.626255	-2.042840	0.719042
10	6	0	4.962689	-1.762932	-1.277226
11	1	0	3.910452	-1.020213	-3.021024
12	6	0	4.863307	-2.119234	0.058741
13	1	0	5.735939	-2.455428	0.599737
14	16	0	3.528212	-2.533342	2.427308
15	8	0	2.826966	-1.440862	3.150545
16	8	0	2.767516	-3.789123	2.480162
17	8	0	4.913904	-2.681769	2.888988
18	16	0	6.526949	-1.854310	-2.126665
19	8	0	6.287606	-2.660201	-3.337607
20	8	0	6.906902	-0.467695	-2.453603
21	8	0	7.468646	-2.493384	-1.194057
22	8	0	0.019588	-1.037528	3.309721
23	1	0	0.956745	-1.169810	3.062709
24	1	0	-0.471724	-1.040462	2.463495
25	6	0	-2.410209	-1.979922	-0.874041
26	6	0	-3.460229	-2.664492	-1.474942
27	6	0	-2.577575	-1.366312	0.419824
28	6	0	-4.704554	-2.754394	-0.831028
29	1	0	-3.306873	-3.119875	-2.447297
30	6	0	-3.843359	-1.461645	1.035632
31	6	0	-4.891025	-2.155759	0.403065
32	1	0	-5.852342	-2.210220	0.891363
33	8	0	-1.514092	-0.752888	0.901031
34	16	0	-4.205168	-0.782453	2.652740
35	8	0	-5.670602	-0.804666	2.773467
36	8	0	-3.549927	-1.660340	3.634791
37	8	0	-3.677542	0.598290	2.688115
38	16	0	-6.037313	-3.630300	-1.626113
39	8	0	-6.290885	-2.933241	-2.900350
40	8	0	-5.555421	-5.007655	-1.836969
41	8	0	-7.183868	-3.564355	-0.706287
42	6	0	0.211803	2.337409	-0.103127
43	6	0	-0.766389	2.226827	-1.152572
44	6	0	0.533731	3.622928	0.377539
45	6	0	-1.391359	3.364918	-1.650168
46	6	0	-0.112962	4.758292	-0.138290
47	6	0	-1.067476	4.632846	-1.135255
48	1	0	-2.126814	3.262872	-2.441054
49	1	0	0.144694	5.733925	0.246630
50	8	0	-0.999627	0.996881	-1.600724
51	16	0	1.830063	3.867656	1.579629
52	8	0	3.087864	3.463995	0.931630
53	8	0	1.803337	5.299970	1.913256
54	8	0	1.509086	3.027240	2.754138

55	16	0	-1.906981	6.072255	-1.765674
56	8	0	-1.267545	7.236634	-1.131798
57	8	0	-1.726696	6.049752	-3.228822
58	8	0	-3.324379	5.928019	-1.384042
59	8	0	-1.442594	1.167038	4.320917
60	1	0	-0.820629	0.469432	4.030495
61	1	0	-2.242756	0.981885	3.787906
62	8	0	3.398451	1.240731	3.858513
63	1	0	3.220703	0.335210	3.541442
64	1	0	2.741111	1.812206	3.412334
65	8	0	0.487115	0.802180	-3.966697
66	8	0	0.276902	-2.883535	-3.454837
67	1	0	0.915099	-2.207164	-3.161737
68	1	0	-0.438937	-2.696437	-2.807931
69	1	0	1.078968	0.227206	-3.441579
70	1	0	-0.201750	0.971857	-3.290692

E(UTPSSh) = -6532.9543959 Hartree
Zero-point correction = 0.400056
Thermal correction to Energy = 0.457409
Thermal correction to Enthalpy = 0.458354
Thermal correction to Gibbs Free Energy = 0.302053
Sum of electronic and zero-point Energies = -6532.554340
Sum of electronic and thermal Energies = -6532.496986
Sum of electronic and thermal Enthalpies = -6532.496042
Sum of electronic and thermal Free Energies = -6532.652343

Table S2. Optimized Cartesian coordinates (Å) of the $[Fe(\textit{Tiron})_2(H_2O)_2]^{5-}\cdot 9H_2O$ system obtained with DFT calculations (0 Imaginary Frequencies).

Center Number	Atomic Number		Coordinates (Angstroms) X	Y	Z
1	8	0	1.919939	1.082018	-1.180797
2	8	0	-0.640093	2.321065	-1.426573
3	1	0	-0.730246	3.229873	-1.041883
4	8	0	-0.735770	-0.633602	-1.197947
5	8	0	1.357346	-0.433830	0.835996
6	8	0	0.570847	2.401890	1.267777
7	1	0	-0.274128	2.763516	1.625855
8	1	0	1.089326	3.176899	0.950771
9	1	0	-1.507639	2.154716	-1.876375
10	8	0	1.781464	4.637595	0.196995
11	8	0	2.650788	3.504534	-2.044869
12	1	0	2.185079	4.312732	-0.655787
13	1	0	2.451198	5.160573	0.651116
14	1	0	2.361738	2.579505	-1.808613
15	1	0	2.107355	3.758720	-2.799654
16	8	0	-1.785323	3.604203	2.036008
17	1	0	-2.578127	3.009291	1.963232
18	1	0	-1.863804	4.059223	2.881894
19	8	0	-4.818588	3.035577	-0.760886
20	1	0	-5.717223	2.721962	-0.913920
21	1	0	-4.577879	2.702333	0.138590
22	8	0	-3.059722	2.150372	-2.642787
23	1	0	-3.723544	2.463408	-1.977731
24	1	0	-3.080620	2.805109	-3.350175

25	8	0	-0.951073	4.824693	-0.344513
26	1	0	-1.348466	4.559539	0.510488
27	1	0	-0.016688	5.015889	-0.130423
28	26	0	0.181680	0.806569	-0.136601
29	6	0	2.942439	0.374398	-0.670729
30	6	0	2.608367	-0.462843	0.435577
31	6	0	4.243179	0.411331	-1.154222
32	6	0	3.608403	-1.267499	1.004698
33	6	0	5.232475	-0.381758	-0.552012
34	1	0	4.488390	1.052862	-1.992915
35	6	0	4.916668	-1.218611	0.510762
36	1	0	5.679827	-1.837617	0.959907
37	16	0	3.192848	-2.388558	2.324772
38	8	0	2.603043	-1.576799	3.406669
39	8	0	2.223755	-3.347930	1.759229
40	8	0	4.447954	-3.029099	2.727101
41	16	0	6.907751	-0.285837	-1.162566
42	8	0	6.838772	-0.619241	-2.594948
43	8	0	7.340140	1.103466	-0.934074
44	8	0	7.682559	-1.260797	-0.382059
45	8	0	-0.101195	-0.739705	3.531497
46	1	0	0.820244	-1.011407	3.345111
47	1	0	-0.416073	-0.372970	2.692201
48	6	0	-1.995596	-0.840328	-0.798768
49	6	0	-2.890547	-1.676421	-1.453625
50	6	0	-2.393457	-0.134214	0.380244
51	6	0	-4.204137	-1.801602	-0.978618
52	1	0	-2.570474	-2.214341	-2.338572
53	6	0	-3.730304	-0.233945	0.801911
54	6	0	-4.624480	-1.075091	0.126349
55	1	0	-5.649256	-1.141056	0.460775
56	8	0	-1.464071	0.596312	0.967432
57	16	0	-4.348230	0.779978	2.129378
58	8	0	-5.792046	0.553381	2.177732
59	8	0	-3.649542	0.430759	3.364284
60	8	0	-4.045493	2.189820	1.715130
61	16	0	-5.332140	-2.907736	-1.811938
62	8	0	-5.295072	-2.530175	-3.234072
63	8	0	-4.809547	-4.264876	-1.580521
64	8	0	-6.647242	-2.689049	-1.193192
65	8	0	0.593967	-3.196389	-0.567816
66	1	0	1.137224	-3.122177	0.240588
67	1	0	0.249826	-2.298544	-0.718754
68	8	0	0.817687	-0.302131	-3.525624
69	1	0	1.371358	0.252159	-2.949278
70	1	0	0.119318	-0.573528	-2.899954

E(UTPSSh) = -5362.784478 Hartree

Zero-point correction = 0.458118
Thermal correction to Energy = 0.516085
Thermal correction to Enthalpy = 0.517030
Thermal correction to Gibbs Free Energy = 0.361140
Sum of electronic and zero-point Energies = -5362.326360
Sum of electronic and thermal Energies = -5362.268393
Sum of electronic and thermal Enthalpies = -5362.267448
Sum of electronic and thermal Free Energies = -5362.423338

Table S3. Optimized Cartesian coordinates (Å) of the $[Fe(\textit{Tiron})(H_2O)_4]^{-}\cdot 13H_2O$ system obtained with DFT calculations (0 Imaginary Frequencies).

Center Number	Atomic Number		Coordinates (Angstroms) X	Y	Z
1	8	0	0.508525	-1.721920	-0.418413
2	8	0	-2.150230	-2.470553	0.464008
3	1	0	-2.988178	-2.765230	0.011200
4	8	0	-0.902266	-0.301663	1.826950
5	1	0	-1.257835	0.580192	2.112034
6	8	0	0.033205	0.786257	-0.660284
7	8	0	-1.905103	-1.054691	-2.141947
8	1	0	-2.880135	-1.004601	-2.281580
9	8	0	-2.975126	0.346716	0.016241
10	1	0	-2.949788	1.344654	-0.015836
11	1	0	-1.553627	-1.810184	-2.693641
12	1	0	-2.313377	-2.500352	1.433427
13	1	0	-0.018617	-0.413000	2.249972
14	1	0	-3.447478	0.119619	0.852315
15	8	0	-1.005240	-3.137314	-3.524164
16	8	0	0.546854	-4.191301	-1.571162
17	1	0	-0.424207	-3.611566	-2.876114
18	1	0	-0.433924	-2.905350	-4.265562
19	1	0	0.573602	-3.362143	-1.036868
20	1	0	0.099312	-4.841252	-1.017032
21	8	0	-4.621846	-1.098573	-2.524836
22	1	0	-5.189314	-0.338358	-2.227284
23	1	0	-4.882721	-1.289771	-3.433518
24	8	0	1.849457	2.294456	3.348055
25	1	0	1.687716	1.338523	3.340598
26	1	0	1.676780	2.594604	2.433912
27	8	0	1.317721	-0.644007	3.344369
28	1	0	0.830675	-1.196804	3.990525
29	1	0	2.049841	-1.185906	3.026217
30	8	0	-2.152621	1.979301	2.637118
31	1	0	-2.964249	1.466864	2.805946
32	1	0	-2.359190	2.506268	1.840818
33	8	0	-2.860767	3.012066	0.051541
34	1	0	-2.194242	3.177805	-0.671019
35	1	0	-3.725514	3.311118	-0.290993
36	8	0	-4.069387	-0.125016	2.494807
37	1	0	-3.677504	-0.956002	2.848835
38	1	0	-5.022929	-0.196126	2.618717
39	8	0	-2.744441	-2.445376	3.185385
40	1	0	-1.970589	-2.331067	3.779465
41	1	0	-3.240157	-3.195694	3.535658
42	8	0	-4.428321	-3.181378	-0.735040
43	1	0	-4.605064	-2.495086	-1.418296
44	1	0	-4.374835	-4.016655	-1.214099
45	26	0	-1.181657	-0.739328	-0.228708
46	6	0	1.579921	-0.938828	-0.495895
47	6	0	1.310100	0.463072	-0.583164
48	6	0	2.889256	-1.422828	-0.483198
49	6	0	2.400756	1.359972	-0.548738
50	6	0	3.944344	-0.520000	-0.510757
51	1	0	3.077256	-2.487455	-0.429912
52	6	0	3.698480	0.856801	-0.522975
53	1	0	4.523030	1.554747	-0.481705
54	16	0	2.186803	3.134775	-0.407101

55	8	0	1.560130	3.607404	-1.656338
56	8	0	1.309309	3.329908	0.760463
57	8	0	3.530620	3.678328	-0.219449
58	16	0	5.634852	-1.108280	-0.541786
59	8	0	6.368735	-0.257513	0.404295
60	8	0	5.572007	-2.516861	-0.133098
61	8	0	6.082464	-0.931163	-1.931439
62	8	0	-1.073449	2.990668	-1.922312
63	1	0	-0.749040	2.118577	-1.613275
64	1	0	-0.234030	3.494785	-1.860572
65	8	0	-0.506318	-2.134930	4.884959
66	1	0	-0.758915	-1.678251	5.698858
67	1	0	-0.186374	-3.000274	5.173823
68	8	0	-6.247445	0.851018	-1.642680
69	1	0	-7.052333	0.929318	-2.167806
70	1	0	-5.944662	1.770163	-1.493865
71	8	0	-5.416905	3.498128	-1.076749
72	1	0	-5.379021	4.065373	-1.858319
73	1	0	-6.057387	3.922056	-0.490311

```
---------------------------------------------------------------
E(UTPSSh) = -4269.036793 Hartree
 Zero-point correction = 0.541812
 Thermal correction to Energy = 0.603569
 Thermal correction to Enthalpy = 0.604514
 Thermal correction to Gibbs Free Energy = 0.437815
 Sum of electronic and zero-point Energies = -4268.494981
 Sum of electronic and thermal Energies = -4268.433224
 Sum of electronic and thermal Enthalpies = -4268.432279
 Sum of electronic and thermal Free Energies = -4268.598978
```

References

(1) Baranyai, Z.; Pálinkás, Z.; Uggeri, F.; Maiocchi, A.; Aime, S.; Brücher, E. Dissociation Kinetics of Open-Chain and Macrocyclic Gadolinium(III)-Aminopolycarboxylate Complexes Related to Magnetic Resonance Imaging: Catalytic Effect of Endogenous Ligands. *Chem. - Eur. J.* **2012**, *18*, 16426–16435.

(2) Baranyai, Z.; Brücher, E.; Uggeri, F.; Maiocchi, A.; Tóth, I.; Andrási, M.; Gáspár, A.; Zékány, L.; Aime, S. The Role of Equilibrium and Kinetic Properties in the Dissociation of Gd[DTPA-Bis(Methylamide)] (Omniscan) at near to Physiological Conditions. *Chem. - Eur. J.* **2015**, *21*, 4789–4799.
